# Rictor/mTORC2 signalling contributes to renal vascular endothelial‐to‐mesenchymal transition and renal allograft interstitial fibrosis by regulating BNIP3‐mediated mitophagy

**DOI:** 10.1002/ctm2.1686

**Published:** 2024-05-20

**Authors:** Dengyuan Feng, Zeping Gui, Zhen Xu, Jianjian Zhang, Bin Ni, Zijie Wang, Jiawen Liu, Shuang Fei, Hao Chen, Li Sun, Min Gu, Ruoyun Tan

**Affiliations:** ^1^ Department of Urology the First Affiliated Hospital of Nanjing Medical University Nanjing China; ^2^ Department of Urology the Second Affiliated Hospital of Nanjing Medical University Nanjing China; ^3^ Department of Urology The Affiliated Taizhou People's Hospital of Nanjing Medical University Taizhou China

**Keywords:** mitophagy, renal allograft interstitial fibrosis, Rictor/mTORC2, vascular endothelial cells

## Abstract

**Background:**

Renal allograft interstitial fibrosis/tubular atrophy (IF/TA) constitutes the principal histopathological characteristic of chronic allograft dysfunction (CAD) in kidney‐transplanted patients. While renal vascular endothelial‐mesenchymal transition (EndMT) has been verified as an important contributing factor to IF/TA in CAD patients, its underlying mechanisms remain obscure. Through single‐cell transcriptomic analysis, we identified Rictor as a potential pivotal mediator for EndMT. This investigation sought to elucidate the role of Rictor/mTORC2 signalling in the pathogenesis of renal allograft interstitial fibrosis and the associated mechanisms.

**Methods:**

The influence of the Rictor/mTOR2 pathway on renal vascular EndMT and renal allograft fibrosis was investigated by cell experiments and *Rictor* depletion in renal allogeneic transplantation mice models. Subsequently, a series of assays were conducted to explore the underlying mechanisms of the enhanced mitophagy and the ameliorated EndMT resulting from Rictor knockout.

**Results:**

Our findings revealed a significant activation of the Rictor/mTORC2 signalling in CAD patients and allogeneic kidney transplanted mice. The suppression of Rictor/mTORC2 signalling alleviated TNFα‐induced EndMT in HUVECs. Moreover, *Rictor* knockout in endothelial cells remarkably ameliorated renal vascular EndMT and allograft interstitial fibrosis in allogeneic kidney transplanted mice. Mechanistically, *Rictor* knockout resulted in an augmented BNIP3‐mediated mitophagy in endothelial cells. Furthermore, Rictor/mTORC2 facilitated the MARCH5‐mediated degradation of BNIP3 at the K130 site through K48‐linked ubiquitination, thereby regulating mitophagy activity. Subsequent experiments also demonstrated that BNIP3 knockdown nearly reversed the enhanced mitophagy and mitigated EndMT and allograft interstitial fibrosis induced by *Rictor* knockout.

**Conclusions:**

Consequently, our study underscores Rictor/mTORC2 signalling as a critical mediator of renal vascular EndMT and allograft interstitial fibrosis progression, exerting its impact through regulating BNIP3‐mediated mitophagy. This insight unveils a potential therapeutic target for mitigating renal allograft interstitial fibrosis.

## Graphical abstract

Suppression of Rictor/mTORC2 signalling alleviates EndMT and renal allograft interstitial fibrosis. Activation of the Rictor/mTORC2 signalling pathway promotes the MARCH5‐mediated degradation of BNIP3, thus hindering BNIP3‐mediated mitophagy, and ultimately leading to the development of renal vascular EndMT and renal allograft interstitial fibrosis. Pharmaceutical intervention on inhibiting Rictor/mTORC2 signalling is a potential target for mitigating allograft fibrosis.

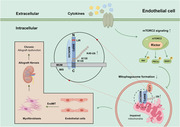

## 1 INTRODUCTION

Kidney transplantation stands as the optimal treatment for the majority of chronic kidney failure patients.^1^ Although short‐term outcomes after kidney transplantation have markedly improved, achieving long‐term survival remains a formidable challenge.^2^ Chronic renal allograft dysfunction (CAD) in kidney transplanted patients, induced by various immunological and nonimmunological injuries, is the main contributor to allograft loss.^3^ CAD is mainly histologically defined by renal interstitial fibrosis and tubular atrophy (IF/TA),^4^ which contributes to the disruption of tissue structure and microperfusion culminating in renal allograft failure.^5^ Myofibroblasts, pivotal in renal allograft interstitial fibrosis, originate from diverse cell types including resident mesenchymal cells and vascular endothelial cells.^6^ Subjected to severe or persistent injuries, renal vascular endothelial cells could progressively transform into myofibroblasts, secreting extracellular matrix (ECM); the process termed as an endothelial‐to‐mesenchymal transition (EndMT).^7^ Throughout EndMT, cells undergo a gradual loss of endothelial characteristics, adopting myofibroblastic traits^8^ influenced by various inflammatory mediators such as TGF‐β and TNFα.^9^ Xu‐Dubois et al. first reported the presence of EndMT in transplanted kidneys and demonstrated its close association with antibody‐mediated rejection, renal graft dysfunction, and proteinuria.^10^ Our previous research has elucidated that EndMT accelerates renal allograft interstitial fibrosis and CAD via the TGF‐β signalling pathway^11^ and in response to TNFα stimulation.^12^ Additionally, TNFα intensified TGF‐β signals, amplifying TGF‐β‐induced EndMT.^13^ Despite these insights, the pathophysiology of EndMT is still not fully comprehended. The unraveling novel molecular pathway implicated in EndMT development is crucial for identifying potential therapeutic targets to alleviate renal allograft interstitial fibrosis.

The mammalian target of rapamycin (mTOR) orchestrates cellular processes including growth, proliferation, and metabolism. It is assembled into two different protein complexes: mTOR Complex 1 (mTORC1) and mTORC2.^14^ Extensive research has implicated mTORC1 signalling in the development of fibrosis across various kidney diseases.^15^, ^16^, ^17^ mTORC2 is comprised of Rictor, mTOR kinase, Protor, mLST8, DEPTOR, and mSIN1.^14^ Within this complex, Rictor serves as a scaffold protein, enabling mTORC2 assembly and facilitating its interaction with regulators and substrates. The absence of Rictor results in diminished mTORC2 signalling. Predominantly, mTORC2 exerts its biological function through phosphorylating members of the AGC (PKA/PKG/PKC) kinase family.^14^ Previous studies reported that TGF‐β1 stimulation activated the mTORC2 signalling pathway, positioning mTORC2 as a vital downstream component of the TGFβ1 pathway.^18^, ^19^ Prior research has demonstrated that Rictor deficiency in macrophages hampers macrophage proliferation and polarization, thereby decelerating renal fibrosis.^20^ Additionally, activation of Rictor/mTORC2 signalling in fibroblast enhances TGFβ1‐induced fibroblast activation, promoting the progression of renal fibrosis.^18^ In this study, we propose that Rictor/mTORC2 signalling maybe play an indispensable role in the occurrence and progression of renal vascular EndMT and renal allograft interstitial fibrosis.

Mitophagy, a specific form of selective autophagy targeting mitochondria, plays a crucial role in eliminating damaged or depolarized mitochondria, thereby upholding mitochondrial quality control and cellular homeostasis.^21^ This process exerts a protective effect in a variety of kidney pathophysiological processes,^22^ including acute kidney injury,^23^ diabetic nephropathy,^24^ and tubulointerstitial fibrosis.^25^ Li Shu et al.^26^ demonstrated that mice with impaired mitophagy due to *PINK1* or *PARK2* gene knockout exhibited more pronounced kidney fibrosis compared with control subjects. Recent study has identified mTORC2 as a negative regulator of mitophagy via the mTORC2‐AKT‐SGK axis.^27^ The knockout of *Rictor* or *SGK1* gene in mouse hepatocytes was found to enhance autophagy and mitophagy by influencing VDAC1 regulation.^28^ Therefore, our research aims to unravel the role of Rictor/mTORC2 in renal allograft interstitial fibrosis, with a specific focus on mitophagy.

In this investigation, we observed a marked upregulation of Rictor/mTORC2 signalling within renal vascular endothelial cells of both CAD patients and mice. Mice engineered with a specific deletion of *Rictor* gene in their endothelial cells demonstrated a reduction in EndMT and a lessened degree of interstitial fibrosis in allogeneic transplanted kidney tissues. On a mechanistic level, the absence of Rictor amplified mitophagy, attributed to a decrease in the proteasome‐depended degradation of BNIP3, resulting in an amelioration of renal vascular EndMT and renal allograft interstitial fibrosis. These findings suggest that Rictor/mTORC2 could be a promising therapeutic target to impede renal allograft interstitial fibrosis and CAD progression.

## 2 METHODS

### 2.1 Patients and tissue samples

We enrolled 20 allogeneic kidney transplanted patients diagnosed with CAD at the First Affiliated Hospital of Nanjing Medical University, all of whom had received a pathological confirmation of renal allograft interstitial fibrosis via nephrectomy or biopsy of the transplanted kidneys. We procured normal kidney tissues from specimens obtained during surgical nephrectomy, ensuring they were located at least 5 cm from the margin of the tumour. Our study protocols were approved by the Ethics Committee of the First Affiliated Hospital of Nanjing Medical University and followed the guidelines of the Declaration of Helsinki and Istanbul. All participants signed informed consent. Table 1 presents the demographic characteristics of participants in the Normal and CAD groups.

**TABLE 1:** Baseline characteristics of the normal and CAD groups.

Clinical variables	Normal group	CAD group	*p‐*value
Case number (*n*)	20	20	>0.05
Gender (male/female)	14/6	12/8	>0.05
Age (years, mean ± SEM)	65.15 ± 1.36	59.60 ± 2.44	>0.05
BMI (kg/m^2^, mean ± SEM)	22.77 ± 0.36	23.51 ± 0.38	>0.05
Transplant duration (years, mean ± SEM)	/	9.76 ± 0.42	/
Primary/secondary transplant	/	20/0	/
PRA before renal transplant (%)	/	0	/
Donor source			/
Living‐related	/	4	
Cadaveric	/	16	
Immunosuppressive regimen			/
Prednisone + MMF + Tac	/	15	
Prednisone + MMF + CsA	/	5	
Serum creatinine (mmol/L, mean ± SEM)	70.55 ± 3.04	435.40 ± 8.43	<0.001

Abbreviations: CAD, chronic allograft dysfunction; CsA, cyclosporine A; MMF, mycophenolate mofetil; PRA, panel reaction antibody; SEM, standard error of the mean; Tac, tacrolimus.

### 2.2 Animals

*Rictor^flox/flox^* mice and *Tie2‐Cre* mice (C57BL/6 background) were generously provided by Prof. Chunsun Dai (The Second Affiliated Hospital of Nanjing Medical University) and Prof. Aihua Zhang (Nanjing Medical University), respectively. Hybridization of *Rictor^flox/flox^* and *Tie2‐Cre* mice created offspring by specifically deleting the *Rictor* gene in endothelial cells (Genotype: *Rictor ^fl/fl^, Cre^+/−^*). The other littermates with the genotype *Rictor ^fl/fl^, Cre^−/−^* served as controls. The primer for genotyping was as follows:

Rictor genotyping: sense:5′‐GACACTGGATTACAGTGGCTTG‐3′

anti‐sense: 5′‐GCTGGGCCATCTGAATAACTTC‐3′

*Tie2*‐Cre genotyping, sense: 5′‐ATTTGCCTGCATTACCGGTC‐3′

anti‐sense: 5′‐ATCAACGTTTTCTTTTCGG‐3′.

Additionally, C57BL/6 and BALB/C mice were sourced from the Animal Center of Nanjing Medical University. All animals were housed in the specific pathogen‐free Laboratory Animal Center at Nanjing Medical University.

### 2.3 Renal allogeneic transplantion mice models

All animal experiments were approved by the Animal Research Ethics Committee of Nanjing Medical University. Renal allogeneic transplantation mice models were constructed as described below. Mice were anesthetised with isoflurane inhalation (RWD Life Science), and the right kidney of the recipient mouse was excised in situ. Subsequently, the left kidney from donor mouse was subsequently transplanted into the recipient mouse. The average total ischemia time during the surgery ranged from 40 to 60 min. BALB/C mice were used as recipients and syngeneic donors (Syn group), whereas C57BL/6 mice were applied as allogeneic donors to create chronic renal allograft interstitial fibrosis mice models (Allo group). Contralateral nephrectomies on the recipient mice were performed on day 7 after kidney transplantation. Tacrolimus (Astellas Pharmaceutical) was administered intraperitoneally at 1.5 mg/kg q.d. for the first 7 days after transplantation and 1.5 mg/kg q.w. for the next 4 weeks. At 16 weeks post‐transplantation, mice were sacrificed humanely, and the harvested transplanted kidneys were immediately fixed in paraffin or stored in liquid nitrogen.

Likewise, BALB/C mice receiving donor kidneys from *Rictor^fl/fl^ Cre^+/−^* or *Rictor^fl/fl^ Cre^−/−^* C57BL/6 mice were designated as the Rictor^−/−^ Allo group or the WT Allo group, respectively. Two weeks after the resection of the left native kidneys in recipient mice, tamoxifen (cat: T5648, Sigma‐Aldrich) was intraperitoneally injected for 5 days at 60 mg/kg to induce *Rictor* gene ablation.

### 2.4 Adeno‐associated virus infection in vivo

For in vivo reduction of BNIP3 expression, AAV9 vectors expressing a shRNA targeting BNIP3 (AAV‐shBNIP3) or a control shRNA (AAV‐shNC) were generated by Jiangsu KeyGEN BioTECH to achieve BNIP3 depletion. Each mouse received a tail intravenous injection of the adenoassociated virus (AAV) vector with a final titer of 1 × 10^12^ viral genome, in the third week after kidney transplantation.

### 2.5 Single‐cell transcriptomic analysis

The profiles of GSE195718 and GSE131882 were obtained from the GEO database (www.ncbi.nlm.nih.gov/geoprofiles/). Uniform manifold approximation and projection (UMAP) was used for dimension reduction. The identification of hub genes was performed using Cytoscape software v3.10.1. Kyoto Encyclopedia of Genes and Genomes (KEGG) pathway and Gene Ontology (GO) enrichment analysis were conducted using DAVID (https://david.ncifcrf.gov/tools.jsp).^29^, ^30^

### 2.6 Cell cultures and treatment

Human Umbilical Vein Endothelial Cells (HUVECs), obtained from Jiangsu KeyGEN BioTECH (KeyGEN, KG419), were cultured in Ham's F‐12K medium (Gibco, 21127030) with 1% Endothelial Cell Growth Supplement (ECGS) (KeyGEN, KGY1052), 1% penicillin‐streptomycin, 10% fetal bovine serum (Gibco, 10099141), and .1 mg/mL heparin (KeyGEN, KGY3149H) at 37°C in a humidified atmosphere containing 5% CO_2_. After being seeded on appropriate culture plates overnight, HUVECs were exposed to 100 ng/mL tumour necrosis factor‐α (TNFα) (Novoprotein, C008) for different time periods.

*Rictor* gene knockout of HUVECs and corresponding control cells were generated using the CRISPR/Cas9 system obtained from Hanbio biotechnology. *Rictor* gene knockout of HUVECs were confirmed by sequencing and validated by western blotting. For Rictor overexpression, HUVECs were transfected with either Empty vector lentivirus or Flag‐Rictor lentivirus (Genechem) following the manufacturer's instruction.

### 2.7 Plasmids, small interfering RNA and reagents

The BNIP3‐HA (P29482), HA‐UB‐K48 (P31802), Myc‐UB‐K48 (P44473), HA‐UB‐K63 (P31800), and Rictor (P41727) plasmids were obtained from MIAOLING BIOLOGY. HA‐UB‐WT, HA‐UB‐K63R, and HA‐UB‐K48R plasmids were sourced from Addgene. HA‐tagged site‐directed mutations of BNIP3 (K130R, K135R) were generated by Jiangsu KeyGEN BioTECH. His‐tagged MARCH5 plasmid was obtained from Beijing Tsingke Biotech Company. All the constructed plasmids were confirmed by DNA sequencing. The plasmids transfection was performed using Lipofectamine 3000 (Invitrogen, L3000008).

The small interfering RNA (siRNAs) were synthesized by Jiangsu KeyGEN BioTECH and transfected using RFect siRNA Transfection Reagent (Baidai biotechnology, 11013). The targeted siRNA sequences were shown as follows:

siRictor: 5′‐GCAGACUCUAUGCAACAAATT‐3′

siBNIP3: 5′‐GAACUGCACUUCAGCAAUAAUTT‐3′

siMARCH5: 5′‐GGUUGUAGGUCAUAAAGAATT‐3′

CCCP (S6494), Mdivi‐1 (S7162), MG132 (S2619), CHX (S7418), and CQ (S6999) were purchased from Selleck.

### 2.8 Western blotting and IP assays

Cells or tissues were lysed using lysis buffer (Fdbio science, FD009) supplemented with 1 mM phenylmethylsulfonyl fluoride (Fdbio science, FD0100) as well as a protease and phosphatase inhibitors cocktail (ThermoFisher Scientific, 78442). The mitochondrial protein of HUVECs was isolated and extracted using a mitochondrial protein extraction kit (BestBio). Protein concentration was quantified with a BCA protein quantitative kit (Beyotime, P0010).

For western blotting assays, proteins were separated by SDS‐polyacrylamide gel electrophoresis and transferred to PVDF membranes (Millipore, ISEQ00010). After blocking in 5% nonfat milk solution at room temperature for 2 h, the membranes were incubated with the primary antibodies at 4°C overnight, then with the HRP‐conjugated secondary antibodies at room temperature for 2 h and detected by ECL reagent (Biosharp, BL520A).

For immunoprecipitation (IP) assays, cells were lysated using IP lysis buffer (ThermoFisher Scientific, 87787) containing 1 mM PMSF. Protein samples were incubated with the primary antibodies at 4°C overnight and then mixed with Protein A/G magnetic beads (Bimake, B23201) at room temperature for 30 min. For proteins tagged with HA or Flag, protein lysates were directly incubated with anti‐HA (Bimake, B26201) or anti‐Flag (Bimake, B26101) magnetic beads at 4°C overnight. After magnetic separation, the protein‐beads complexes were washed with lysis buffer three times, then boiled in 1X SDS loading buffer and subjected to western blotting analysis.

The primary antibodies and secondary antibodies used in our research are listed in Table S1.

### 2.9 Ubiquitination assays

To evaluate the ubiquitination of endogenous BNIP3, we immunoprecipitated BNIP3 using anti‐BNIP3 antibody and analyzed it by western blot assays with anti‐Ub antibody. For the analysis of BNIP3 ubiquitination, HUVECs were transfected with plasmids expressing the target genes. The whole cell lysates were immunoprecipitated with indicated antibodies or magnetic beads and subjected to western blotting assays.

### 2.10 Cycloheximide chase assays

Cycloheximide (CHX) chase assays were performed to determine the half‐lives of proteins. Cells were treated with 200 μg/mL CHX at different time points, and then cell proteins were extracted to analyze BNIP3 levels by western blotting assays.

### 2.11 Transmission electron microscopy analysis

Preparation of HUVECs for transmission electron microscopy (TEM) analysis was as described.^31^ The prepared electron microscope samples were processed and scanned using TEM (FEI Tecnai G2) at the Analysis and Testing Center, Nanjing Medical University.

### 2.12 RNA extraction and reverse‐transcriptase PCR assay

Total RNA was isolated from cell lines using the RNA‐Quick Purification Kit (Shanghai YISHAN biotechnology, RN001) and reverse transcribed with the HiScript II Q RT SuperMix (Vazyme, R222‐01). The qPCR was carried out using the ChamQ SYBR qPCR Master Mix (Vazyme, Q341‐02) on the StepOnePlus Real‐Time PCR system (Applied Biosystems). The specific primer sequences used were as follows:

BNIP3: forward: 5′‐CAGGGCTCCTGGGTAGAACT‐3′, reverse: 5′‐CTACTCCGTCCAGACTCATGC‐3′

GAPDH: forward: 5′‐GGAGCGAGATCCCTCCAAAAT‐3′, reverse: 5′‐ GGCTGTTGTCATACTTCTCATGG‐3′

### 2.13 Renal allograft histological pathology staining analyses

Renal allograft histological pathology analyses were evaluated by Masson trichrome staining and hematoxylin and eosin (H&E) as previously described.^31^ HE staining was utilized to assess allograft structure changes, while Masson staining was employed to assess renal interstitial fibrosis degree.

### 2.14 Indirect immunofluorescence and immunohistochemical staining assays

After renal tissues were fixed and embedded, renal sections underwent indirect immunofluorescence (IF) and immunohistochemical (IHC) staining. IHC staining of tissues was performed with the HRP Kit (CWbio, CW2069S). In the case of IF staining, renal sections were initially baked at 65°C for 2 h. Subsequently, they were deparaffinized with xylene, rehydrated through graded ethanol solutions (100%, 100%, 95%, 85%, and 75%, respectively), and rinsed with distilled water. Following this, the renal sections were undergone antigen retrieval using an enhanced citrate solution (Beyotime, P0083). Subsequently, they were incubated with 3% hydrogen peroxide and then blocked with a Blocking Buffer (Beyotime, P0260). Subsequent to these steps, the renal sections were incubated with the primary antibodies at 4°C overnight, and then with the secondary antibodies at room temperature for 1 h. Visualization of nuclei was achieved using DAPI Fluoromount‐G (SouthernBiotech, 0100−20).

In preparation for cells’ IF staining, cells were seeded onto glass coverslips at 30% confluence. Following fixation of the cells using 4% formalin, permeabilization, and blocking with Blocking Buffer (Beyotime, P0260), the cell samples were incubated with target protein antibodies at 4°C overnight. Following that, the coverslips were incubated with fluorescence‐conjugated secondary antibodies and stained with DAPI Fluoromount‐G (SouthernBiotech, 0100−20). To label the mitochondria, the cells were incubated with MitoTracker Red CMXRos (Thermo Fisher, M7512), followed by fixation and immunostaining using the specified antibody. Images were captured either through an Olympus optical microscope or a Zeiss LSM 880 NLO confocal microscope.

The primary antibodies and secondary antibodies used for this study are listed in Table S1.

### 2.15 ELISA assay

The levels of TNFα in serum of the Syn and Allo groups were assessed with Mouse TNFα ELISA kit (Elabscience, E‐EL‐M3063) according to the manufacturer's instructions.

### 2.16 Statistical analysis

The data were presented as mean ± SEM and each experiment was repeated independently at least three times. Student's *t*‐test was applied to determine statistical differences between the two groups, while one‐way ANOVA followed by Dunnett or Tukey post hoc tests were used for statistical comparisons among multiple groups. The GraphPad Prism Software 8.0 was used for statistical analyses. A *P*‐value of <0.05 was considered statistically significant.

## 3 RESULTS

### 3.1 The upregulation of Rictor in allogeneic renal vascular endothelial cells of CAD patients is unveiled through single‐cell transcriptomic analysis

**FIGURE 1:**
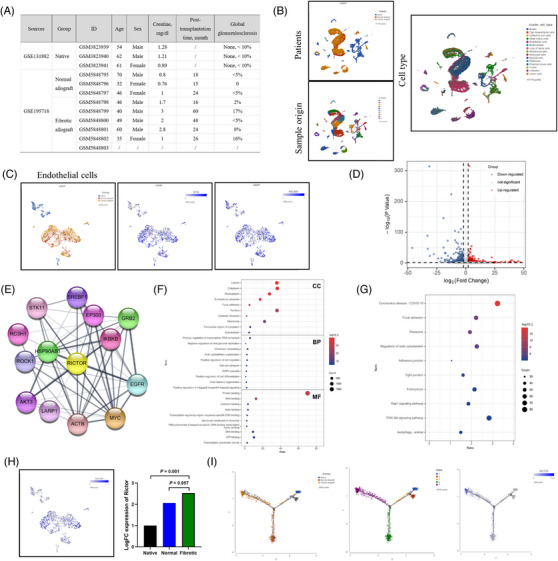
The upregulation of Rictor in allogeneic renal vascular endothelial cells of CAD patients was unveiled through single‐cell transcriptomic analysis. (A) The information on patients and renal tissues for single‐cell RNA sequencing, including healthy adult kidney tissue samples from three individuals (native group), normal renal allograft tissues from three stable renal transplanted patients (normal allograft group), and fibrotic renal allograft tissues from six CAD patients (fibrotic allograft group). (B) Uniform manifold approximation and projection (UMAP) visualization of cell subclusters from three groups. Different cell subclusters are differently color‐coded. (C) UMAP shows the expressions of classical markers for endothelial cells (PECAM1 and CD34). (D) Volcano plot showing the differential expressed genes (DEGs) in endothelial cells between the native and fibrotic allograft groups, with 1771 upregulated genes and 1008 downregulated genes. (E) Graphical visualization of hub genes and their protein–protein interaction analysis. (F, G) Gene ontology (GO) analysis (F) and Kyoto Encyclopedia of Genes and Genomes (KEGG) analysis (G) of DEGs in endothelial cells between the native and fibrotic allograft group. (H) UMAP shows the scaled expression of Rictor in endothelial cells. (I) Pseudotime analysis showing dynamic changes of Rictor expression in endothelial cells from kidneys in three groups.

To delineate potential key genes involved in the progression of renal vascular EndMT and CAD progression, we systematically analyzed publicly available GSE195718 and GSE131882 datasets. A comprehensive summary of the included datasets is presented in Figure 1A. Patients were categorized into three groups: the native group (comprising healthy adult kidney samples from three individuals), the normal allograft group (consisting of normal renal allograft tissues from three stable kidney transplanted patients), and the fibrotic allograft group (encompassing fibrotic renal allograft tissues from six CAD patients). Subsequently, we acquired 44716 cells from 12 patients for single‐cell RNA sequencing (scRNA‐seq) analysis. UMAP analysis identified all major cell types within the kidney tissues based on the expression of lineage‐specific markers (Figure 1B). EndMT has been established as an important contributor to the development of renal allograft fibrosis.^11^ Therefore, our focus was on the differential expressed genes (DEGs) in endothelial cells, identified using PECAM1 (CD31) and CD34 markers (Figure 1C). We identified 2779 genes comprising 1771 upregulated genes and 1008 downregulated genes between the native and fibrotic allograft groups (Figure 1D). Hub genes in endothelial cells, including Rictor, were illustrated in Figure 1E. GO analysis for the DEGs in endothelial cells revealed enrichment in focal adhesion, actin cytoskeleton organization, actin filament organization, cell‐cell adhesion, and actin filament organization (Figure 1F). KEGG analysis depicted an upregulated pathway including focal adhesion, regulation of adherents junction, actin cytoskeleton, and tight junction (Figure 1G). Subsequently, we analyzed DEGs across the three groups and found that Rictor was highly expressed in PECAM1‐positive endothelial cells in fibrotic allograft group compared to the native group, with its expression in the fibrotic allograft group exhibiting an increasing trend compared to the normal allograft group (Figure 1H). Pseudotime analysis further validated a significant elevation in Rictor expression in endothelial cells along a trajectory from normal allograft tissues to fibrotic allograft tissues (Figure 1I). These findings robustly supported the important role of Rictor in the pathogenesis of EndMT and renal allograft fibrosis.

### 3.2 Rictor/mTORC2 signalling is upregulated in renal vascular endothelial cells of CAD patients and mice

**FIGURE 2:**
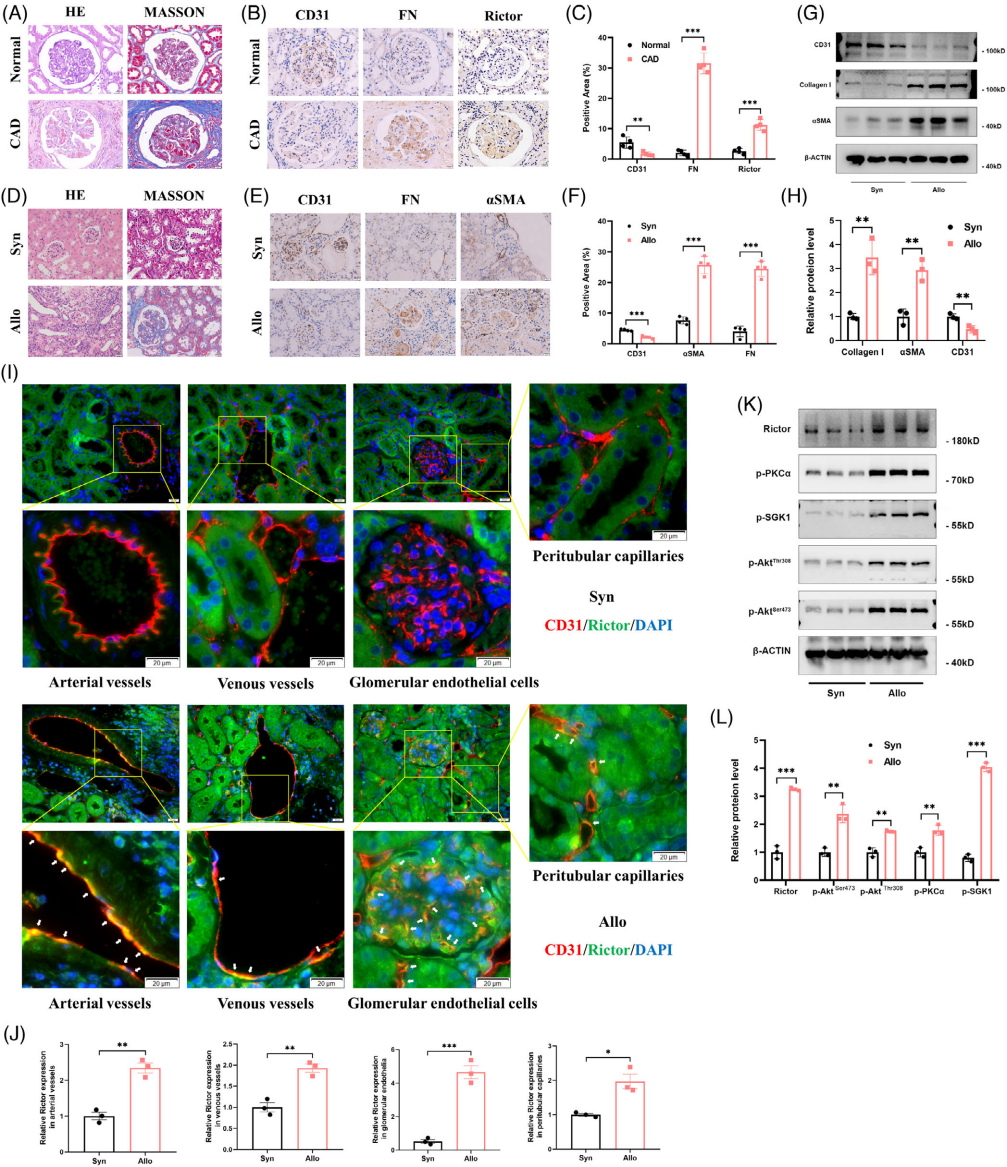
Rictor/mTORC2 signalling is activated in renal vascular endothelial cells from CAD patients and renal allogeneic transplanted mice. (A) Representative images of HE and Masson staining in renal allograft tissues from normal and CAD patients. (B, C) Representative IHC images (B) and statistical graphs of semi‐quantitative analyses (C) of CD31, FN, and Rictor expression in renal allograft tissues from the normal and CAD patients. (*n* = 4; bar = 20 μm). (D) Representative images of HE and Masson staining in transplanted kidney tissues from the Syn and Allo groups. (E, F) Representative IHC images (E) and statistical graphs of semi‐quantitative analyses(F) of CD31, FN, and αSMA expression in transplanted kidney tissues from the Syn and Allo groups. (*n* = 3; bar = 20 μm). (G, H) The results of western blot analyses (G) and quantitative analyses of the relative abundance (H) of CD31, Collagen I, and αSMA expression in transplanted kidney tissues from the Syn (*n* = 3) and Allo (*n* = 3) groups. (I) Representative images of colocalization of CD31 and Rictor (White arrows) in transplanted kidney tissues from the Syn and Allo groups by indirect immunofluorescence double staining (*n* = 3, bar = 20 μm). (J) Result of semi‐quantitative analysis of Rictor expression in transplanted kidney tissues from the Syn and Allo groups by indirect immunofluorescence staining. (K, L) The results of western blot analyses (K) and quantitative analyses of the relative abundance (L) of Rictor, p‐PKCα, p‐SGK1, p‐Akt^Thr308^, and p‐Akt^Ser473^ expression in transplanted kidney tissues from the Syn (*n* = 3) and Allo (*n* = 3) groups. *p < 0.05, **p < 0.01, ***p < 0.001.

To explore the potential involvement of the Rictor/mTORC2 pathway in renal allograft interstitial fibrosis, we initially analyzed Rictor expression in renal allograft tissues from normal individuals (normal group) and CAD patients (CAD group). The results of histological pathology staining indicated disordered renal structure and significant interstitial fibrosis in the CAD group (Figure 2A). Furthermore, IHC assays revealed heightened expression of FN and Rictor, accompanied by downregulation of CD31 expression in the CAD group (Figure 2B, C).

In addition, we generated an allogeneic kidney transplanted mice model to confirm the involvement of Rictor/mTORC2 signalling in the development of renal allograft fibrosis. BALB/C mice received kidneys from either BALB/C or C57BL/6 mice were designated as the Syn (syngeneic) or Allo (allogeneic) group, respectively. The renal allografts of the Allo group displayed interstitial inflammatory cell infiltration, glomerulosclerosis, and significant interstitial fibrosis, as revealed by HE and Masson staining (Figure 2D). Moreover, IHC staining showed increased expressions of FN and αSMA, along with decreased CD31 expression in the Allo group (Figure 2E, F). These findings were confirmed by western blotting assays of Collagen I, αSMA, and CD31 (Figure 2G, H), suggesting the presence of reanl vascular EndMT and interstitial fibrosis in the Allo group. Furthermore, increased Rictor expressions in renal vascular endothelial cells from the Allo group were detected via IF assay (Figure 2I, J). Consistently, remarkable inductions of Rictor, p‐Akt^Ser473^, p‐Akt^Thr308^, p‐PKCα, and p‐SGK1 were detected in kidney tissues from the Allo group (Figure 2K, L), suggesting the activation of the mTORC2 signalling pathway.

In our previous study, TNFα was conclusively identified as a contributor to EndMT.^12^ IHC assays revealed an augmented expression of TNFα in both the CAD group and Allo group, compared to the control and Syn group (Figure S1A–D). ELISA assay also demonstrated a significant elevation in TNFα protein levels in the serum of the Allo group in comparison to the Syn group (Figure S1E). Correspondingly, a decreased CD31 expression and elevated expressions of FSP‐1 and FN were observed in HUVECs following TNF‐α treatment (Figure S1F, G). Besides, the media of the Rictor/mTORC2 signalling pathway were activated in HUVECs treated with TNFα at different time points (Figure S2A, B). Collectively, these findings indicate that Rictor/mTORC2 signalling is upregulated in vascular endothelial cells from the fibrotic kidneys with allograft transplantation, and may be associated with development of renal vascular EndMT.

### 3.3 Activation of Rictor/mTORC2 signalling mediates EndMT formation

**FIGURE 3:**
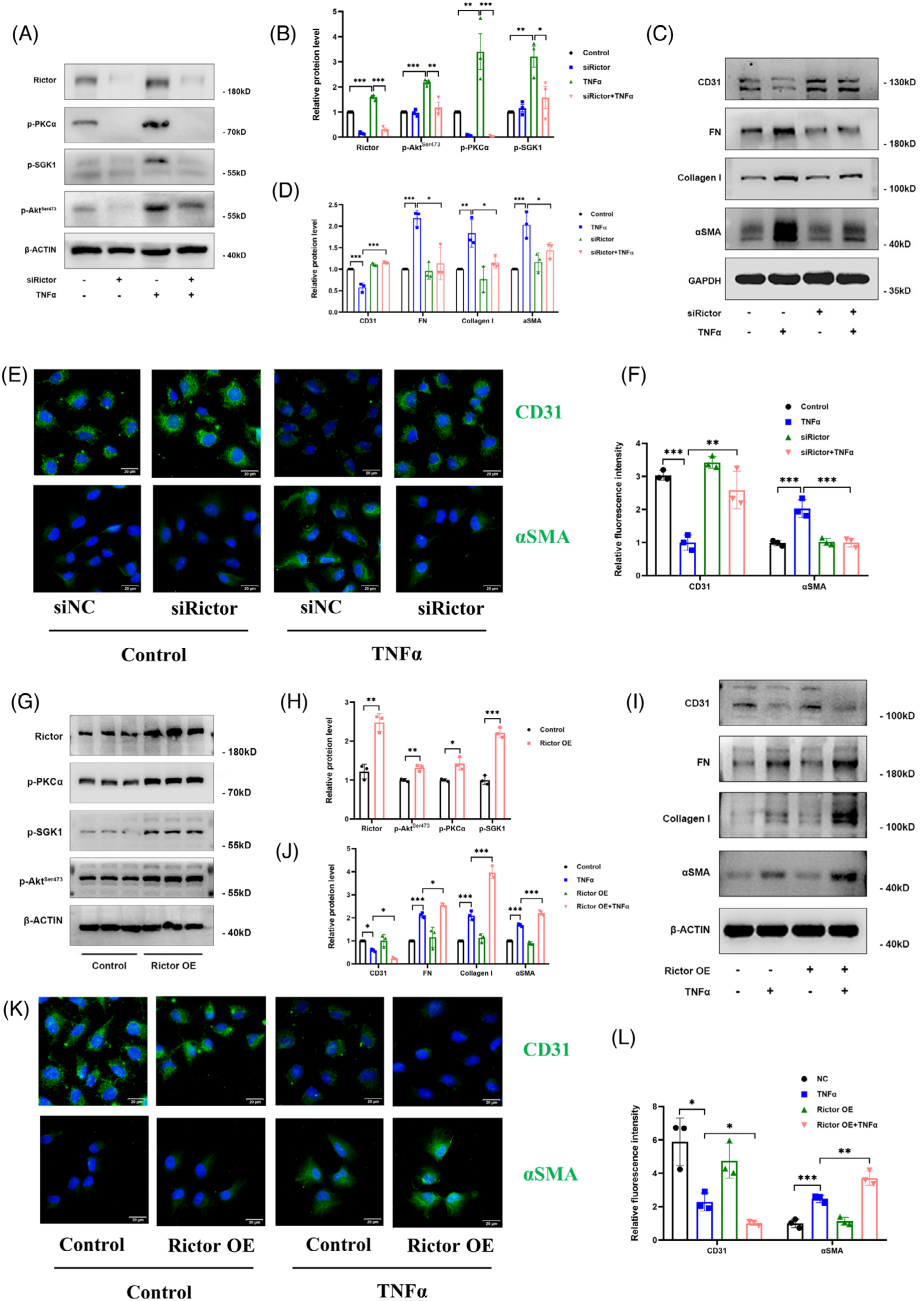
Effects of changes of Rictor/mTORC2 signalling activity to EndMT induced by TNFα in HUVECs. HUVECs were transfected with siNC or siRictor for 24 h. After transfection, part of HUVECs were treated with 100 ng/mL; TNFα for 48 h. (A, B) The results of western blot analyses (A) and quantitative analyses of the relative abundance (B) of Rictor, p‐PKCα, p‐SGK1, p‐Akt^Thr308^, and p‐Akt^Ser473^ expression in HUVECs transfected with siNC or siRictor. (C, D) The results of western blot analyses (C) and quantitative analyses of the relative abundance (D) of CD31, FN, Collagen I, and αSMA expression in HUVECs treated by TNFα or not after transfected with siNC or siRictor. (E, F) Representative images (E) and statistical graphs of semi‐quantitative analyses (F) for IF staining of CD31 and αSMA expression in HUVECs treated by TNFα or not after transfected with siNC or siRictor (bar = 20 μm). HUVECs were transfected with control or Rictor plasmids for 24 h. After transfection, HUVECs were treated with 100 ng/mL TNFα for 48 h. (G, H) The results of western blot analyses (G) and quantitative analyses of the relative abundance (H) of Rictor, p‐PKCα, p‐SGK1, and p‐Akt^Ser473^ expression in HUVECs transfected with control or Rictor plasmids. (I, J) The results of western blot analyses (I) and quantitative analyses of the relative abundance (J) of CD31, FN, Collagen I, and αSMA expression in HUVECs treated by TNFα or not after transfected with control or Rictor plasmids. (K, L) Representative images (K) and statistical graphs of semi‐quantitative analyses (L) for IF staining of CD31 and αSMA expression in HUVECs treated by TNFα or not after transfected with control or Rictor plasmids (bar = 20 μm). *p < 0.05, **p < 0.01, ***p < 0.001.

To elucidate the influence of the Rictor/mTORC2 pathway on EndMT, Rictor siRNAs were applied to reduce Rictor expression in HUVECs, followed by stimulation with 100 ng/mL TNFα for 48 h. Subsequent analyses revealed a significant reduction in Rictor expression and notable downregulation of mTORC2 signalling media in HUVECs transfected with Rictor siRNA compared with control siRNA under TNFα treatment (Figure 3A, B). As depicted in Figure 3C, D, TNFα treatment resulted in upregulation of FN, Collagen I, and αSMA expression, alongside a decrease in CD31 expression, all of which were effectively reversed by Rictor siRNA. These changes at the protein level were also corroborated by IF staining assays (Figure 3E, F). In a subsequent experiment, we transfected HUVECs with either control or Rictor overexpression plasmids for 24 h, followed by TNFα treatment (100 ng/mL) for 48 h. In line with our previous results, Rictor overexpression in HUVECs activated the mTORC2 pathway and exacerbated TNFα‐induced EndMT, as confirmed by western blotting and IF staining assays (Figure 3G–L). Furthermore, in the human renal glomerular endothelial cells, Rictor knockdown with Rictor siRNA could also attenuate TNFα‐induced EndMT (Figure S3A, B). Collectively, these findings robustly demonstrate that Rictor/mTORC2 signalling has a significant impact on mediating the development of EndMT.

### 3.4 *Rictor* gene knockout could enhance BNIP3‐mediated mitophagy in HUVECs

**FIGURE 4:**
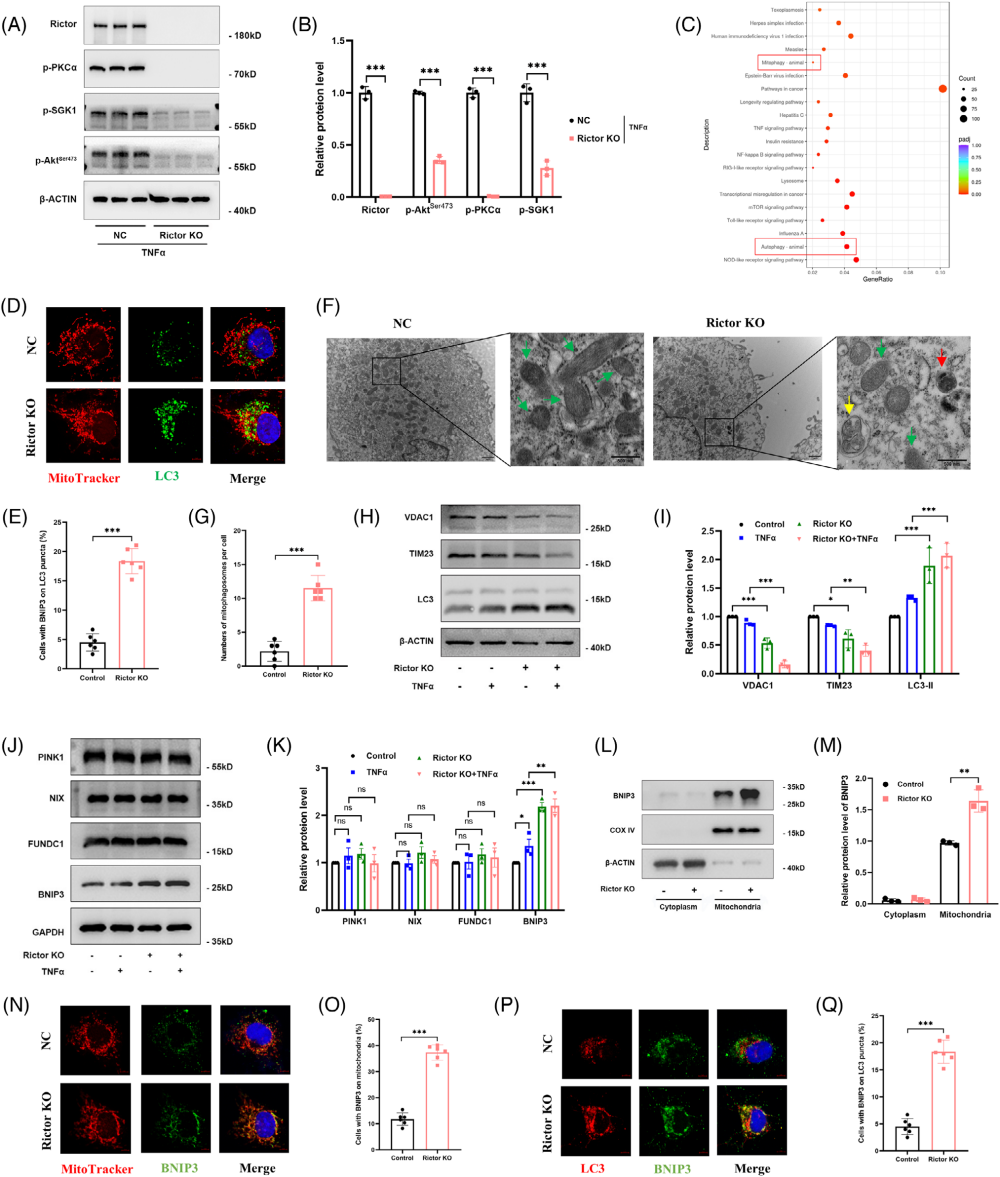
Knockout of *Rictor* gene can activate mitophagy and enhance mitophagy induced by TNFα in BNIP3‐dependent manner in HUVECs. Stable *Rictor* gene knockout (KO) of the HUVEC line was generated using CRISPR/Cas9‐mediated gene editing. (A, B) The results of western blot analyses (A) and quantitative analyses of the relative abundance (B) of Rictor, p‐PKCα, p‐SGK1, and p‐Akt^Ser473^ expression in the control and Rictor KO HUVECs under TNFα treatment. (C) KEGG pathway analysis of differentially expressed genes from the RNA‐seq assay in the control and Rictor KO HUVECs. (D) Representative images of colocalization of mitochondrial marker (MitoTracker) and LC3 expression in HUVECs from the control and Rictor KO group by IF double staining. (*n* = 6; bar = 5 μm). € Result of semi‐quantitative analysis of LC3 expression in HUVECs from the control and Rictor KO group by IF staining. (*n* = 6; bar = 5 μm). (F, G) Representative TEM images (F) and statistical graph of quantitative analysis (G) of mitophagosomes in the control and Rictor KO HUVECs (bar = 1 μm). (Green arrowheads: normal mitochondria; yellow arrowhead: autophagosome; red arrowhead: mitophagosome). (H, I) The results of western blot analyses (H) and quantitative analyses of the relative abundance (I) of VDAC1, TIM23, and LC3 expression in the control and Rictor KO HUVECs treated by 100 ng/mL TNFα or not for 48 h. (J, K) The results of western blot analyses (J) and quantitative analyses of the relative abundance (K) of PINK1, NIX, FUNDC1, and BNIP3 expression in the control and Rictor KO HUVECs treated by 100 ng/mL TNFα or not for 48 h. (L, M) The results of western blotting analysis (L) and quantitative analysis of the relative abundance (M) of BNIP3 expression in the mitochondrial and cytosolic fractions of HUVECs from the control and Rictor KO group. (N–Q) Representative images and statistical graphs of semi‐quantitative analyses of IF double staining‐labeling MitoTracker and BNIP3, LC3, and BNIP3. (*n* = 6, bar = 5 μm). Data were presented as mean ± SEM. *p < 0.05, **p < 0.01, ***p < 0.001.

To assess the potential mechanism of Rictor on EndMT formation, we established a *Rictor* gene knockout (KO) HUVEC line utilizing CRISPR/Cas9‐mediated gene editing (Figure S4A, B) and subsequently confirmed its validity via a western blotting assay (Figure 4A). Additionally, we observed a substantial inactivation of the Rictor/mTORC2 signalling pathway after Rictor knockout under TNFα treatment (Figure 4A). To further explore how Rictor/mTORC2 signalling regulates EndMT progression in endothelial cells, we performed a transcriptomic analysis of Rictor KO HUVECs and control cells. The KEGG pathway analysis of the RNA‐seq data showed significant enrichment of mitophagy‐related genes in Rictor KO HUVECs compared to the controls (Figure 4C). IF staining revealed that colocalization of MitoTracker with LC3, indicating mitophagosome formation, was more prominent in HUVECs after Rictor KO (Figure 4D, E). Transmission electron microscopy (TEM) scan analysis also provided the final piece of evidence, revealing an increased number of mitophagosomes in HUVECs with Rictor KO (Figure 4F, G). Western blotting assay further confirmed that Rictor KO induced a significant increase in LC3 expression, accompanied by a noticeable downregulation of mitochondrial membrane protein VDAC1 and TIMM23, particularly after TNFα co‐stimulation (Figure 4H, I), indicating that Rictor KO could induce mitophagy activation.

Mitophagy, a selective process for eliminating dysfunctional or damaged mitochondria via autophagy, is regulated by two major mitophagy pathways: the PINK1–Parkin pathway and the mitophagy receptors pathway.^32^ In our endeavour to elucidate the mechanisms underlying Rictor's modulation of mitophagy, we assessed several pivotal molecules, including PINK1, NIX, FUNDC1, and BNIP3, all of which were reported to play crucial roles in mitophagy mediation. Western blotting analyses revealed a marked upregulation of BNIP3 expression following *Rictor* gene knockout in HUVECs (Figure 4J, K). Conversely, the expression of PINK1, NIX, and FUNDC1 remained statistically unchanged between the Rictor KO and control groups (Figure 4J, K). Mitochondrial protein extraction results further verified the augmented BNIP3 expression in HUVECs after *Rictor* gene knockout (Figure 4L, M). Additionally, *Rictor* gene knockout in HUVECs resulted in increased colocalization of BNIP3 with LC3‐II and MitoTracker (Figure 4N–Q). Taken together, these findings strongly suggest that Rictor expression depletion could enhance BNIP3‐mediated mitophagy in renal vascular endothelial cells, subsequently attenuating EndMT and renal allograft interstitial fibrosis.

### 3.5 Changes in mitophagy activity and BINP3 expression can influence TNFα‐induced EndMT in HUVECs

**FIGURE 5:**
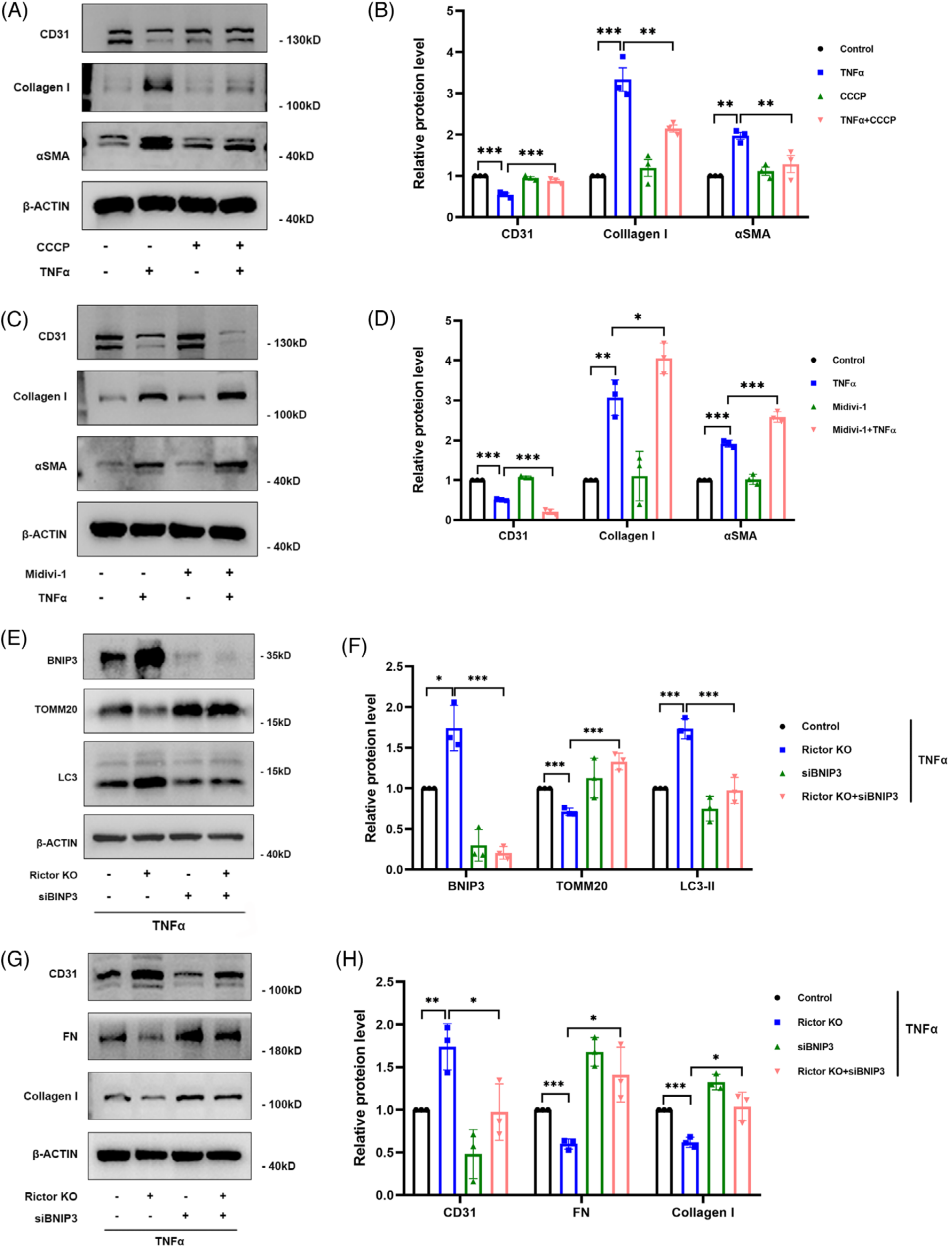
Changes in mitophagy activity and BINP3 expression can influence TNFα‐induced EndMT in HUVECs. HUVECs were pretreated with 20 μM CCCP or control solvent for 2 h and then treated with 100 ng/mL TNFα or control solvent for 24 h. (A, B) The results of western blot analyses (A) and quantitative analyses of the relative abundance (B) of CD31, Collagen I, and αSMA expression in different groups. HUVECs were pretreated with 10 μM Mdivi‐1 or control solvent for 24 h and then treated with 100 ng/mL TNFα or control solvent for 24 h. (C, D) The results of western blot analyses (C) and quantitative analyses of the relative abundance (D) of CD31, Collagen I, and αSMA expression in different groups. HUVECs from the control and Rictor KO groups were transfected with control or BNIP3 siRNA and then treated with 100 ng/mL TNFα for 24 h. (E, F) The results of western blot analysis (E) and quantitative analyses of the relative abundance (F) of BNIP3, TOMM20, and LC3 expression in different groups. (G, H) The results of western blot analysis (G) and quantitative analyses of the relative abundance (H) of CD31, FN, and Collagen I expression in different groups. Data were presented as mean ± SEM. *p < 0.05, **p < 0.01, ***p < 0.001.

To assess the impact of mitophagy activity on TNFα‐induced EndMT, mitophagy agonist (CCCP) and inhibitor (Mdivi‐1) were employed in HUVECs. HUVECs were pretreated with 20 μM CCCP for 2 h, followed by 100 ng/mL TNFα treatment for 24 h. Western blotting analysis demonstrated that CCCP reversed TNFα‐induced upregulation of Collagen I and αSMA expression, and the downregulation of CD31 expression (Figure 5A, B). Additionally, HUVECs were pretreated with 10 μM Mdivi‐1 for 24 h, followed by 100 ng/mL TNFα treatment for 24 h. Figure 5C, D illustrated that Mdivi‐1 treatment exacerbated TNFα‐induced EndMT, as confirmed by western blotting assay.

Subsequently, to further validate the importance of BNIP3‐mediated mitophagy in the mitigation of TNFα‐induced EndMT resulting from Rictor downregulation, BNIP3 siRNA was transfected into the control and Rictor KO groups. In HUVECs, following transfection with BNIP3 siRNA, the Rictor knockout‐induced upregulation of mitophagy was essentially nullified, manifesting as comparable levels of BNIP3, TOMM20, and LC3 expression (Figure 5E, F). Meanwhile, BNIP3 knockdown abrogated the alleviation of EndMT induced by downregulated Rictor expression (Figure 5G, H). In summary, these results strongly suggest that BNIP3‐mediated mitophagy activation could ameliorate TNFα‐induced EndMT in HUVECs.

### 3.6 Rictor/mTORC2 signalling could promote the proteasome depended degradation of BNIP3 at K130 through K48‐linked ubiquitination

**FIGURE 6:**
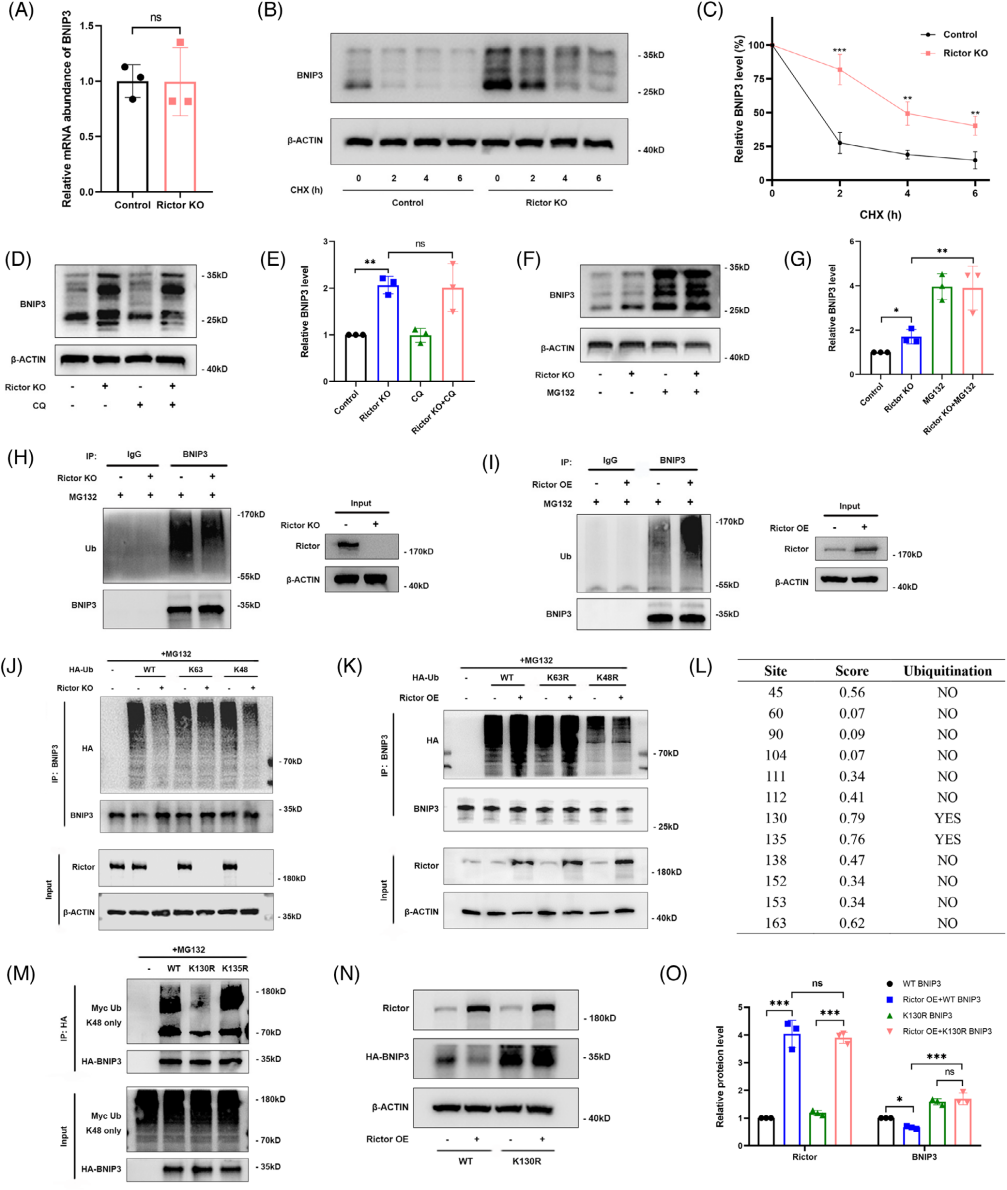
Rictor promotes the proteasome‐dependent degradation of BNIP3 at the K130 site through K48‐linked ubiquitination. (A) RT‐PCR analysis results of BNIP3 mRNA expression in the control and Rictor KO HUVECs. (B, C) The results of cycloheximide (CHX) chase assay for BNIP3 expression in the control and Rictor KO HUVECs treated with CHX (200 μg/mL) for the indicated time points. (D, E) The results of western blot analysis (D) and quantitative analysis of the relative abundance (E) of BNIP3 in the control and Rictor KO HUVECs treated with 10 μM CQ for 24 h. (F, G) The results of western blot analysis (F) and quantitative analysis of the relative abundance (G) of BNIP3 in the control and Rictor KO HUVECs treated with 10 μM MG132 for 6 h. (H) The results of co‐immunoprecipitation assay for the ubiquitination of BNIP3 in the control and Rictor KO HUVECs treated by MG132. (I) The results of co‐immunoprecipitation assay for the ubiquitination of BNIP3 in the control and Rictor overexpression HUVECs treated by MG132. (J) The results of co‐immunoprecipitation assays for the ubiquitination of BNIP3 in the control and Rictor KO HUVECs treated by MG132 after transfection with WT‐BNIP3, K63‐linked BNIP3, or K48‐linked BNIP3 plasmids for 48 h. (K) The results of co‐immunoprecipitation assay for the ubiquitination of BNIP3 in the control and Rictor overexpression HUVECs treated by MG132 after transfection with WT‐BNIP3, K63R‐linked BNIP3, or K48R‐linked BNIP3 plasmids for 48 h. (L) The predictive result of BNIP3 ubiquitination site by using CKSAAP_UbSite. (M) The results of co‐immunoprecipitation assay for the ubiquitination of BNIP3 in HUVECs transfected with Myc‐ubiquitin and different mutant BNIP3 plasmids. (N, O) The results of western blot analysis (N) and quantitative analysis of the relative abundance (O) of Rictor and BNIP3 expression in the control and Rictor overexpression HUVECs transfected with either WT BNIP3 or K130R BNIP3 plasmids. Data were presented as mean ± SEM. *p < 0.05, ***p < 0.001.

To explore how the regulatory mechanism of Rictor on BNIP3, we initially assessed BNIP3 expression at the transcriptional level in HUVECs in the Rictor KO and the control groups, observing comparable BNIP3 mRNA levels in both groups (Figure 6A). Therefore, we hypothesized that Rictor may influence BNIP3 protein degradation rather than its mRNA transcription. To confirm this, we utilized CHX, a well‐established protein synthesis inhibitor, to measure protein half‐lives. Our observation indicated an extension in the half‐life of BNIP3 in HUVECs following Rictor knockout (Figure 6B, C). Furthermore, we discovered that the proteasome inhibitor MG132, but not the autophagy inhibitor CQ, impeded the BNIP3 upregulation induced by Rictor knockout in HUVECs (Figure 6D–G), further supporting the involvement of Rictor in mediating the proteasome depended on degradation of BNIP3. Consistently, Rictor knockout in HUVECs resulted in a reduction in the ubiquitination of endogenous BNIP3 (Figure 6H), while Rictor overexpression led to an increase in the ubiquitination of endogenous BNIP3 (Figure 6I). Mammalian cells harbour a diverse array of ubiquitin chains, each serving specific biological functions. Predominantly, K48‐linked polyubiquitination facilitates proteasome‐dependent degradation of substrate proteins, while K63‐linked polyubiquitination specifically modifies certain proteins, thereby initiating corresponding signal pathways. To ascertain the particular polyubiquitination modification of BNIP3 affected by Rictor, we transfected either K48‐linked or K63‐linked polyubiquitination plasmids into HUVECs. Our results indicated that Rictor knockout led to a decrease in K48‐linked polyubiquitination of BNIP3, leaving K63‐linked polyubiquitination unaffected (Figure 6J). To verify this, we expressed K48‐resistant (K48R) or K63‐resistant (K63R) polyubiquitin chains in Rictor‐overexpressed HUVECs. Consistently, K48R polyubiquitin abolished the augmented ubiquitination of BNIP3 (Figure 6K). These results confirmed that Rictor regulated the proteasome‐dependent degradation of BNIP3 through K48‐linked polyubiquitination. Our subsequent objective was to identify the specific lysine residues on BNIP3 responsible for its ubiquitination and subsequent degradation. Among the 12 lysine residues in human BNIP3 protein, two were predicted as potential ubiquitination sites by CKSAAP_UbSite (http://systbio.cau.edu.cn/cksaap_ubsite/) (Figure 6L). To test these predictions, we created two HA‐tagged BNIP3 mutant plasmids with individual lysine residues substituted by arginine. The following immunoprecipitation assays demonstrated that the K130R mutant showed reduced ubiquitination, suggesting that the lysine residue K130 is crucial for the BNIP3 ubiquitination mediated by Rictor (Figure 6M). Furthermore, the K130 mutant displayed a substantial reduction in BNIP3 degradation mediated by Rictor overexpression (Figure 6N, O). Therefore, these results elucidate that Rictor regulates mitophagy by promoting the K48‐linked ubiquitination of BNIP3 at the K130 site.

### 3.7 MARCH5 is the E3 ubiquitin ligase that specifically regulates the proteasome‐dependent degradation of BNIP3 mediated by Rictor/mTORC2 signalling pathway

**FIGURE 7:**
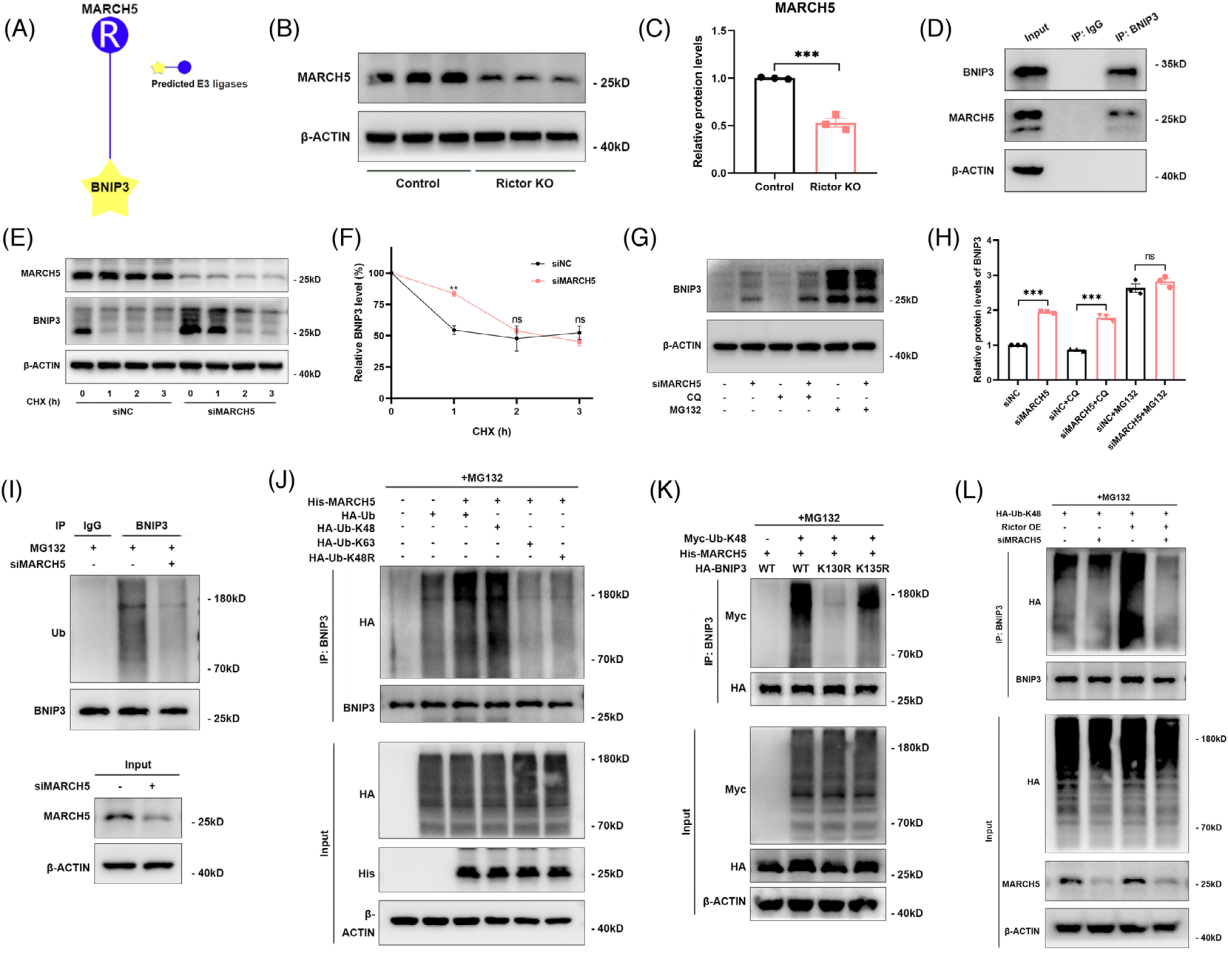
MARCH5 is the E3 ubiquitin ligase that specifically regulates the proteasome‐dependent degradation of BNIP3 mediated by the Rictor/mTORC2 signalling pathway. (A) The predictive result of the E3 ubiquitin ligases interacting with BNIP3 by using the UbiBrowser platform. (B, C) The results of western blot analyses (B) and quantitative analyses of the relative abundance (C) of MARCH5 expression in the control and Rictor KO HUVECs. (D) Co‐immunoprecipitation assay was performed to detect the association between BNIP3 and MARCH5. (E, F) The results of CHX chase assays for BNIP3 expression in HUVECs (transfected with control or MARCH5 siRNAs) treated with CHX (200 μg/mL) for the indicated time points. (G, H) The results of western blot analyses (G) and quantitative analyses of the relative abundance (H) of BNIP3 in siNC or siMARCH5 transfected HUVECs treated with 10 μM CQ for 24 h or 10 μM MG132 for 6 h. (I) The results of co‐immunoprecipitation assays for the ubiquitination of BNIP3 in the siNC or siMARCH5 transfected HUVECs treated by MG132. (J) After transfection of HUVECs with the indicated plasmids for 48 h, co‐immunoprecipitation assay for the ubiquitination of BNIP3 with the indicated antibodies was performed. (K) Co‐immunoprecipitation assay for the ubiquitination of HA‐BNIP3 was performed in HUVECs transfected with the indicated plasmids. (L) After transfection of control or MRACH5 siRNA in the control or Rictor overexpression HUVEECs, co‐immunoprecipitation assay for the ubiquitination of BNIP3 was performed with the indicated antibodies. Data were presented as mean ± SEM. *p < 0.05, **p < 0.01, ***p < 0.001.

To investigate the specific E3 ubiquitin ligase regulating Rictor‐medicated degradation of BNIP3, we used the UbiBrowser platform (http://ubibrowser.ncpsb.org) to predict the ligases interacting with BNIP3 (Figure 7A). According to our predicted results, we screened the MARCH5 protein that was downregulated by Rictor knockout (Figure 7B, C). The following immunoprecipitation assays revealed endogenous interactions between BNIP3 and MARCH5 in HUVECs (Figure 7D), suggesting the specific E3 ubiquitin ligase mediating BNIP3 degradation is MARCH5. The CHX chase assay revealed that MARCH5 knockdown significantly prolongs the half‐life of BNIP3 (Figure 7E, F). We found that it was MG132 and not CQ that could reverse the upregulated BNIP3 caused by MARCH5 knockdown in HUVECs (Figure 7G, H). The ubiquitination assays showed that MARCH5 knockdown decreased BNIP3 ubiquitination (Figure 7I), while MARCH5 overexpression promoted K48‐linked, but not K63‐linked ubiquitination of BNIP3, consistent with the effect of MARCH5 on the degradation of BNIP3 (Figure 7J). To identify the ubiquitinated sites of BNIP3 regulated by MARCH5, the His‐MARCH5 plasmid and HA‐BNIP3 plasmid or other mutants were co‐transfected into HUVECs. The ubiquitination assays indicated that the ubiquitination of BNIP3 in the K130R mutant of BNIP3 was significantly reduced compared with that of wild‐type or K135R mutant (Figure 7K), suggesting the Lys130 site was the ubiquitinated site of BNIP3 mediated by MARCH5. To explore the influence of MARCH5 on the proteasomal degradation of BNIP3 mediated by Rictor, we transfected MARCH5 siRNA into the control or Rictor overexpressed HUVECs. The ubiquitination assays indicated that Rictor overexpression promoted the ubiquitination of BNIP3, which could be reversed by silencing MARCH5 (Figure 7L). All these results demonstrate that MARCH5 acts as the specific E3 ubiquitin ligase regulating proteasome‐dependent degradation of BNIP3 mediated by the Rictor/mTORC2 signalling pathway.

### 3.8 Ablation of *Rictor* gene in endothelial cells can alleviate renal vascular EndMT and allograft interstitial fibrosis

**FIGURE 8:**
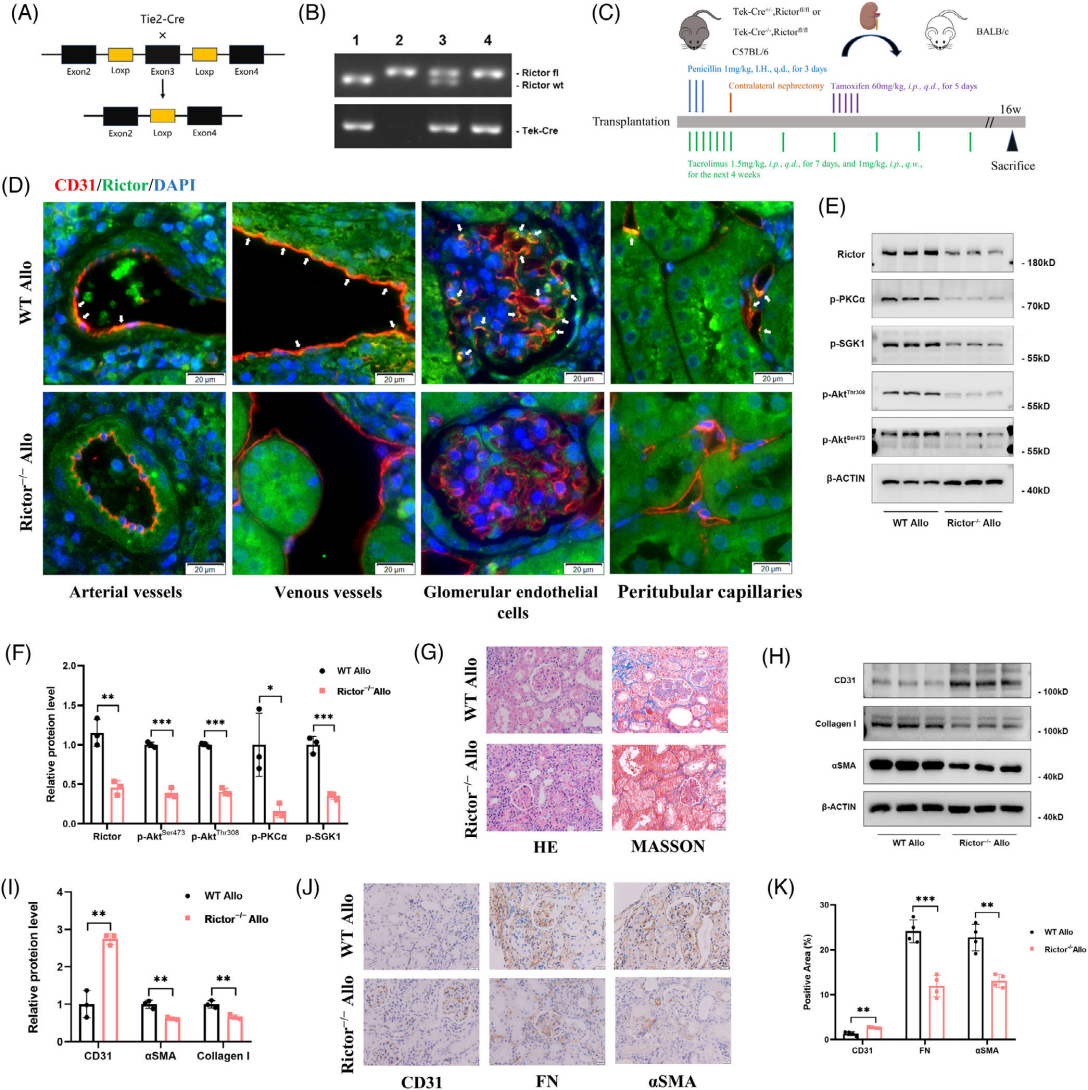
Ablation of *Rictor* gene in endothelial cells alleviates EndMT and renal allograft interstitial fibrosis in renal allogeneic transplanted mice. (A) The diagram illustrates the strategy for generating mice with a specific deletion of *Rictor* gene in endothelial cells. (B) Representative PCR results of genotyping the mice by PCR analysis of genomic DNA. Lane 1: *Rictor ^wt/wt^, Tie2‐ Cre^+/−^*; Lane 2: *Rictor ^fl/fl^, Tie2‐ Cre^−/−^*; *Rictor ^fl/wt^, Tie2‐ Cre^+/−^*; *Rictor ^fl/fl^, Tie2‐ Cre^+/−^*. (C) The schematic diagram for the surgery procedure of mice renal allogeneic transplantation model for the WT Allo and Rictor^−/−^ Allo groups. (D) Representative IF double staining images of colocalization of CD31 and Rictor (White arrows) in transplanted kidney tissues from the WT Allo and Rictor^−/−^ Allo groups (bar = 10 μm). (E, F) The results of western blot analyses (D) and quantitative analyses of the relative abundance (E) of Rictor, p‐PKCα, p‐SGK1, p‐Akt^Thr308^, and p‐Akt^Ser473^ expression in the WT Allo and Rictor^−/−^ Allo groups. (G) Representative images of HE and Masson staining in renal allograft tissues from the WT Allo and Rictor^−/−^ Allo groups. (H, I) The results of western blot analyses (H) and quantitative analyses of the relative abundance (I) of CD31, Collagen I, and αSMA expression in transplanted kidney tissues from the WT Allo and Rictor^−/−^ Allo groups. (J, K) Representative IHC images (I) and statistical graphs of semi‐quantitative analyses (J) of CD31, FN, and αSMA expression in transplanted kidney tissues from the WT Allo and Rictor^−/−^ Allo groups. (*n* = 3, bar = 20 μm). Data were presented as mean ± SEM. *p < 0.05, **p < 0.01, ***p < 0.001.

To further investigate the influence of *Rictor* expression in endothelial cells during the progression of renal allograft interstitial fibrosis, we created the animal model with endothelial cell‐specific *Rictor* gene deletion using the Cre‐LoxP system. The crossbreeding strategy employed to generate the knockout mice is shown in Figure 8A. Subsequently, we established chronic renal allograft interstitial fibrosis mice model, the procedure is shown in Figure 8C. BALB/C mice received donor kidneys from *Rictor ^fl/fl^ Cre^+/−^* (Figure 8B, Lane 4) or *Rictor ^fl/fl^ Cre^−/−^* (Figure 8B, Lane 2) mice were divided into the Rictor^−/−^ Allo group or the WT Allo group, respectively. We employed western blotting analysis and IF staining assays to evaluate Rictor expression and mTORC2 signalling in the transplanted kidneys (Figure 8D–F). The results demonstrated a significant reduction in Rictor protein levels and a substantial suppression of mTORC2 signalling in the Rictor^−/−^ Allo group than the WT Allo group (Figure 8E, F).

We performed HE and Masson staining assays on transplanted kidney tissues from various groups. The Rictor^−/−^ Allo group demonstrated substantial reductions in renal glomerular sclerosis and interstitial ECM deposition (Figure 8G). The fibrotic area in the WT Allo group was significantly larger than that in the Rictor^−/−^ Allo group (Figure 8G). Additionally, we assessed the extent of renal fibrosis and vascular EndMT. The western blotting assay demonstrated that the targeted deletion of Rictor expression in endothelial cells resulted in a reduction in Collagen I and αSMA expression, while concurrently enhancing CD31 expression (Figure 8H, I). IHC staining for FN, αSMA, and CD31 expression provided further confirmation of the western blotting results (Figure 8J, K). In summary, those results underscored the ameliorative impact of Rictor deletion in endothelial cells on renal vascular EndMT and interstitial fibrosis in allogeneic kidney transplanted mice.

### 3.9 *Rictor* gene ablation could enhance BNIP3‐mediated mitophagy in chronic renal allograft interstitial fibrosis mice model

**FIGURE 9:**
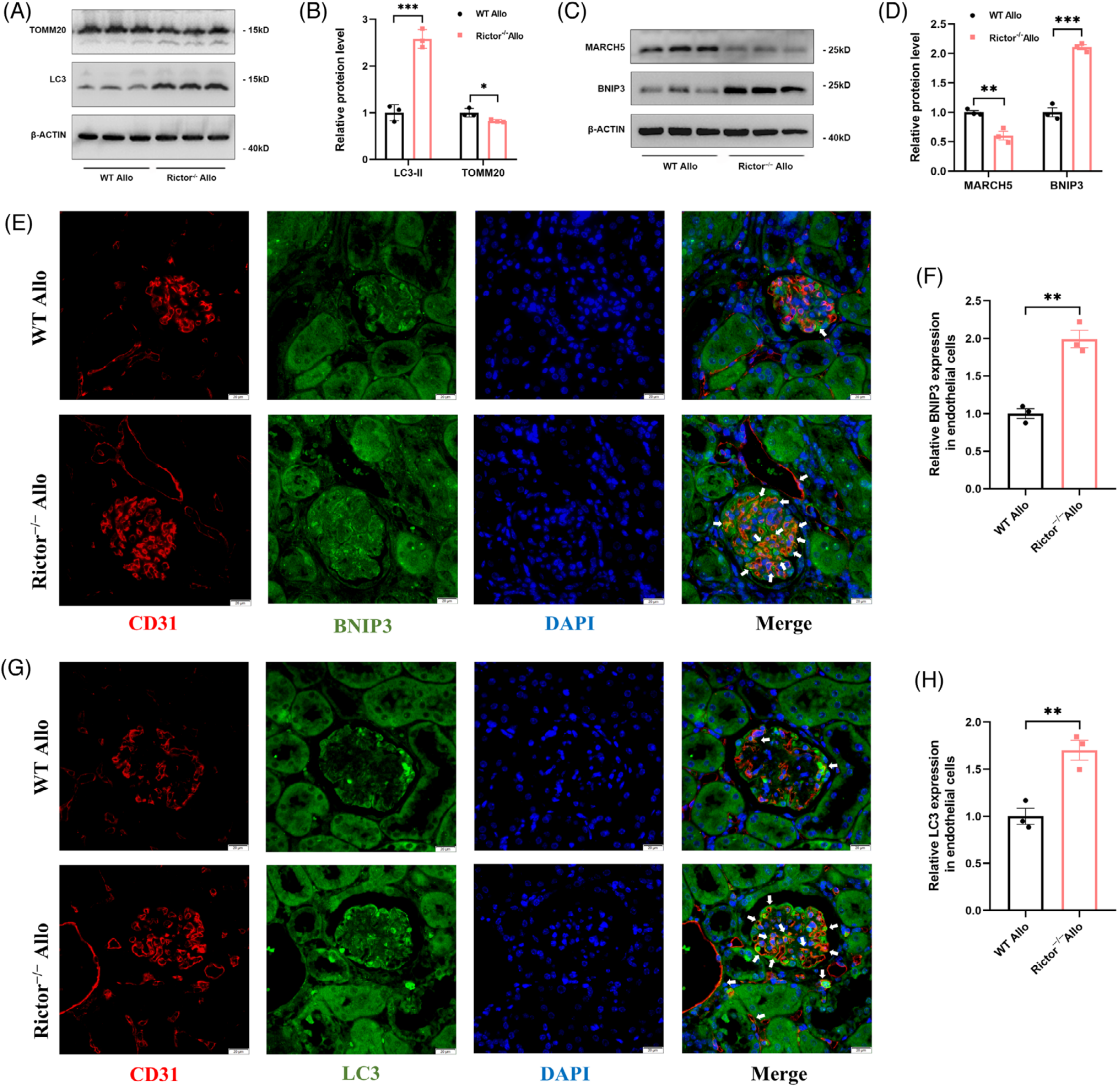
*Rictor* gene ablation activates BNIP3‐mediated mitophagy in renal allogeneic transplanted mice. (A–D) The results of western blot analyses (A, C) and quantitative analyses of the relative abundance (B, D) of TOMM20, LC3, MARCH5, and BNIP3 expression in transplanted kidney tissues from the WT Allo and Rictor^−/−^ Allo groups. (E, G) Representative images of IF double staining‐labeling CD31 and LC3 (E) (White arrows), CD31 and BNIP3 (G) (White arrows) in transplanted kidney tissues from the WT Allo and Rictor^−/−^ Allo groups. (F, H) Statistical graphs of IF staining semi‐quantitative analyses of LC3 (F) and BNIP3 (H) expression in transplanted kidney tissues from the WT Allo and Rictor^−/−^ Allo groups. (*n* = 3; bar = 10 μm). Data were presented as mean ± SEM. *p < 0.05, **p < 0.01, ***p < 0.001.

In chronic renal allograft interstitial fibrosis mice model, we explored the effects of Rictor ablation on mitophagy activity. Elevated LC3 expression in transplanted kidney tissues was observed in the Rictor^−/−^ Allo group, while TOMM20 expression was reduced in the Rictor^−/−^ Allo group compared with the WT Allo group (Figure 9A, B). These findings suggested an augmentation of mitophagy in the Rictor^−/−^ Allo group. Furthermore, Rictor ablation resulted in a significant upregulated BNIP3 expression and downregulated MARCH5 expression (Figure 9C, D). Moreover, the Rictor^−/−^ Allo group exhibited increased colocalization of CD31 with LC3 and BNIP3 (Figure 9E–H), further confirming the upregulated BNIP3‐mediated mitophagy in renal vascular endothelial cells. These findings collectively demonstrate that BNIP3 expression and mitophagy activity are augmented in the renal vascular endothelial cells following Rictor knockout.

### 3.10 Knockdown of BNIP3 reverses mitophagy activation, the improved renal vascular EndMT and interstitial fibrosis caused by *Rictor* gene knockout

**FIGURE 10:**
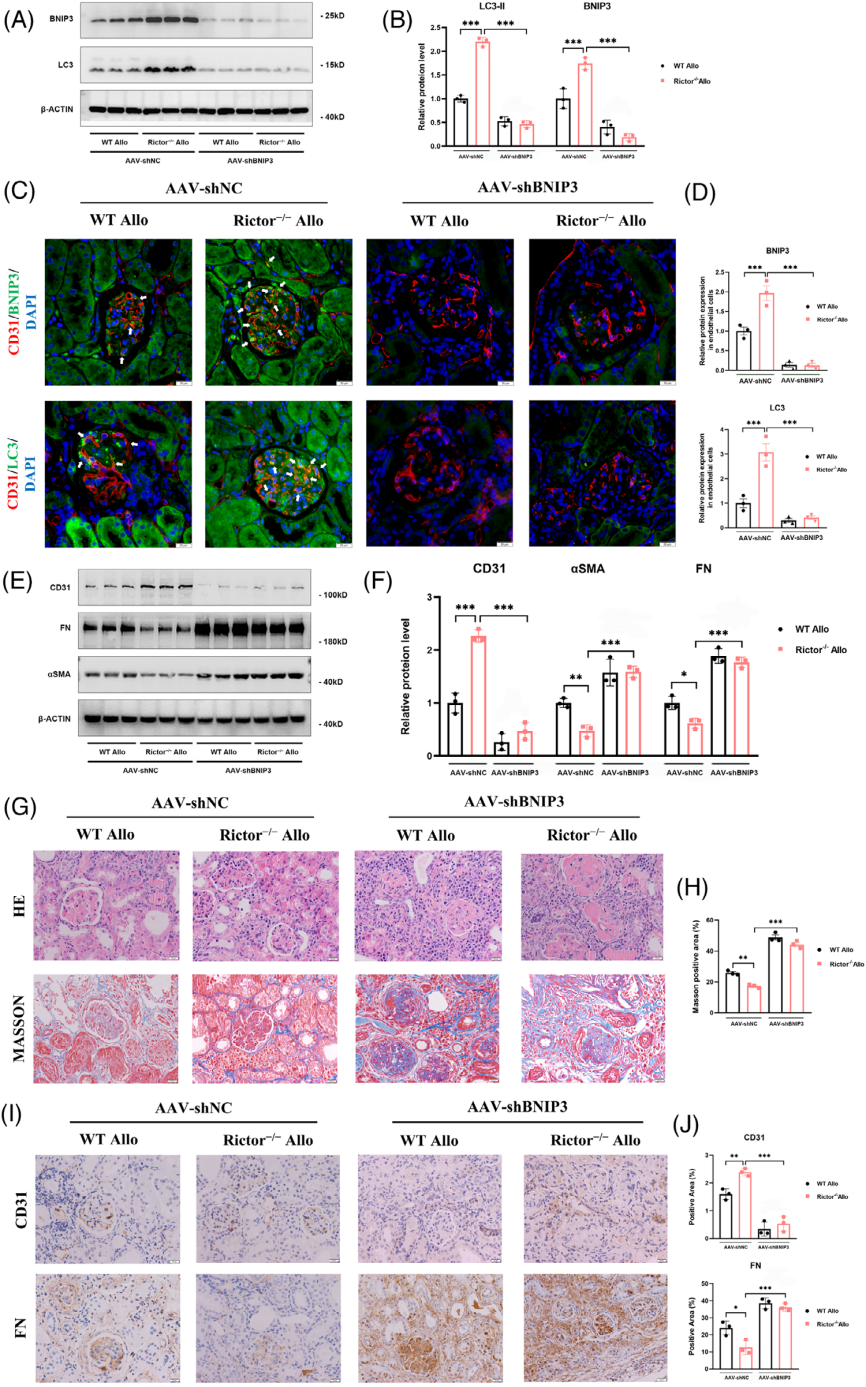
Downregulation of BNIP3 expression reverses mitophagy activation, alleviated EndMT and renal allograft interstitial fibrosis caused by *Rictor* gene ablation in renal allogeneic transplanted mice. Mice from the WT Allo and Rictor^−/−^ Allo groups were injected with adeno‐associated virus (AAV) encoding PHB2 shRNA (AAV‐shBNIP3) or negative control AAV (AAV‐shNC) by tail veins at the third week after kidney transplantation. Transplanted kidney tissues were used for further analyses. (A, B) The results of western blot analysis (A) and quantitative analyses of the relative abundance (B) of BNIP3 and LC3 expression in the transplanted kidney tissues from the WT Allo and Rictor^−/−^ Allo groups injected with AAV‐shBNIP3 or AAV‐shNC. (C) Representative images of IF double staining‐labeling CD31 and BNIP3 (White arrows), CD31 and LC3 (White arrows) in the transplanted kidney tissues from the WT Allo and Rictor^−/−^ Allo groups injected with AAV‐shBNIP3 or AAV‐shNC. (*n* = 3; bar = 10 μm). (D) Statistical graphs of IF staining semi‐quantitative analyses of LC3 and BNIP3 expression in the transplanted kidney tissues from four groups. (E, F) The results of western blot analysis (E) and quantitative analyses of the relative abundance (F) of CD31, FN, and αSMA expression in the transplanted kidney tissues from four groups. (G) Representative images of HE and Masson staining in transplanted kidney tissues from four groups. (H) The results of semi‐quantitative analyses of fibrosis positive area by Masson's staining in different groups. (I, J) Representative IHC images (I) and statistical graphs of semi‐quantitative analyses (J) of CD31 and FN expression in transplanted kidney tissues from four groups. Data were presented as mean ± SEM. *p < 0.05, **p < 0.01, ***p < 0.001.

To delve further into the role of the mitophagy receptor BNIP3 in the enhanced mitophagy and amelioration of EndMT and renal allograft interstitial fibrosis associated with Rictor ablation in endothelial cells, we injected the kidney‐transplanted mice from the WT Allo group or Rictor^−/−^ Allo group with AAV‐shBNIP3 or AAV‐shNC. We found that BNIP3 knockdown substantially negated the effect of Rictor ablation on mitophagy, as evidenced by the diminished expressions of LC3 and BNIP3 in transplanted kidney tissues from both the WT Allo and Rictor^−/−^ Allo groups (Figure 10A, B). In concordance, the knockdown of BNIP3 led to reduced colocalization of CD31 with BNIP3 and LC3, denoting inhibited BNIP3‐mediated mitophagy in endothelial cells within the transplanted kidney tissues (Figure 10C, D).

Moreover, BNIP3 knockdown effectively mitigated the disparities in the expression of EndMT markers, including FN, αSMA, and CD31, observed between transplanted kidney tissues from the WT Allo and Rictor^−/−^ Allo groups (Figure 10E, F). Additional validations were provided through HE, MASSON, and IHC staining assays, which confirmed that BNIP3 knockdown counteracted the ameliorative effects on renal allograft damage and fibrosis attributed to Rictor ablation in endothelial cells (Figure 10G–J). Hence, these results strongly support the hypothesis that inhibition of Rictor/mTORC2 signalling could enhance BNIP3‐mediated mitophagy in renal vascular endothelial cells, which, in turn, alleviates the development of EndMT and renal allograft interstitial fibrosis.

## 4 DISCUSSION

**FIGURE 11:**
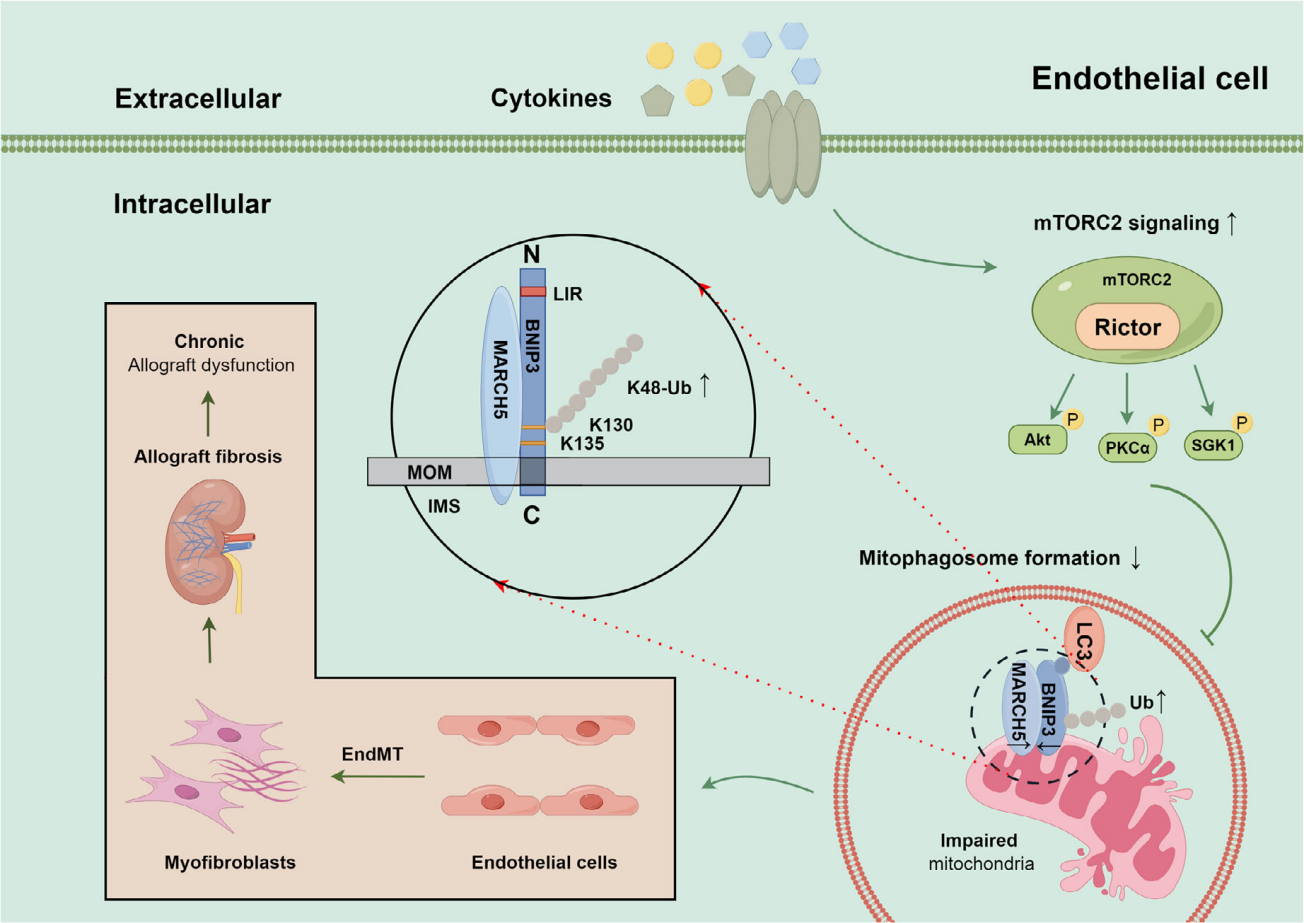
The Rictor/mTORC2 signalling pathway inhibits BNIP3‐mediated mitophagy and subsequently promotes renal vascular EndMT and renal allograft interstitial fibrosis by regulating ubiquitin‐proteasome‐depended degradation of BNIP3 (by Figdraw). Activation of the Rictor/mTORC2 signalling pathway promotes the ubiquitin‐proteasome‐depended degradation of BNIP3, thus hindering BNIP3‐mediated mitophagy, ultimately leading to the development of renal vascular EndMT and renal allograft interstitial fibrosis.

This study underscores the pivotal role that Rictor/mTORC2 signalling plays in the development of renal vascular EndMT and renal allograft interstitial fibrosis. Activation of Rictor/mTORC2 signalling was noted in renal allograft tissues exhibiting interstitial fibrosis. In contrast, its attenuation led to the alleviation of renal vascular EndMT and renal allograft interstitial fibrosis, a process mediated by the enhancement of BNIP3‐dependent mitophagy. Additionally, BNIP3 knockdown negated the benefits of activated mitophagy, and mitigated EndMT and renal fibrosis induced by Rictor knockout. This research elucidates the substantial influence of Rictor/mTORC2 signalling in the pathogenesis of renal allograft interstitial fibrosis and proposes a viable therapeutic strategy for the prevention and treatment of it (Figure 11).

Endothelial cells, as a crucial cell type within renal tissues, function as the intermediary between the recipient and donor, rendering them particularly vulnerable to both immune and non‐immune injuries after kidney transplantation.^33^ Prolonged damage to these cells impairs allograft function, diminishes graft longevity, and facilitates the development of EndMT.^34^ While EndMT has been deemed to play an important role in the pathogenesis of fibrosis, its underlying mechanisms are not yet fully understood.^35^ In our prior research, we established that TGF‐β‐induced renal vascular EndMT in endothelial cells contributed to renal allograft interstitial fibrosis and CAD progression through activating the TGF‐β/Smad and Akt/mTOR/p70S6K signalling pathways.^11^ Similarly, Franzin et al.^36^ reported extracellular vesicles from antibody‐mediated allograft rejection expedited complement activation in endothelial cells, leading to EndMT and early allograft interstitial fibrosis following kidney transplantation. MicroRNAs have also been shown to modulate EndMT progression and allograft interstitial fibrosis by suppressing and degrading targeted mRNA.^37^ In our research, we found that activation of Rictor/mTORC2 signalling could promote the progression of EndMT and allograft interstitial fibrosis after kidney transplantation.

The mTORC1 pathway has been linked to various diseases,^38^, ^39^, ^40^ and its activation has been shown to negatively regulate renal interstitial fibrosis.^16^, ^38^, ^41^ In our previous research, we demonstrated that the mTORC1/p70S6 kinase pathway was upregulated in renal allograft interstitial fibrosis, and inhibited with bortezomib led to a reduction in ECM deposition and mitigation of allograft interstitial fibrosis.^42^ Concurrently, the influence of Rictor/mTORC2 signalling pathways on the progression of fibrotic renal disease has garnered attention. In mice undergoing unilateral ureter obstruction (UUO), conditional deletion of *Rictor* gene in fibroblasts led to a decrease in collagen deposition, cell apoptosis, and inflammatory cell infiltration, an effect which were mediated through the suppression of Akt phosphorylation.^18^ A separate study highlighted that blocking the PKCα signalling downstream of the mTORC2 pathway in fibroblasts could halt fibroblast activation and renal interstitial fibrosis in UUO.^43^ Likewise, deactivating Rictor/mTORC2 signalling in macrophage within UUO mice inhibited multiple profibrotic cytokines, reduced macrophage activation and ameliorated renal interstitial fibrosis.^20^ In the context of diabetic nephropathy, the downregulation of mTORC2 signalling was found to lessen podocyte apoptosis, alleviate glomerular injury, and decrease albuminuria.^44^ Nonetheless, the specific impact of Rictor/mTORC2 signalling pathway on renal allograft interstitial fibrosis necessitates further exploration. In this research, we observed an upregulation of the Rictor/mTORC2 signalling pathway in transplanted kidneys displaying interstitial fibrosis. The downregulation of mTORC2 signalling, achieved via Rictor ablation or knockdown in endothelial cells, led to mitigation of EndMT and renal allograft fibrosis. Previous studies highlight the influence of the Rictor/mTORC2 pathway on renal fibrosis through Akt,^18^ PKCα^43^ signalling, and the Hippo pathway.^19^ Given the intricacies of the downstream signalling regulated by Rictor/mTORC2, additional research is imperative to unravel the principal downstream pathway impacting EndMT and renal fibrosis following kidney transplantation.

This study unveiled a compelling finding that suppression of the Rictor/mTORC2 pathway in endothelial cells could enhance BNIP3‐mediated mitophagy, which in turn mitigates renal vascular EndMT and interstitial fibrosis after kidney transplantation. Mitophagy, a selective autophagy process targeting damaged mitochondria, plays a crucial role in maintaining mitochondrial quality. Nonetheless, the role of mitophagy in renal allograft interstitial fibrosis has been scarcely explored. Our previous research revealed that ATG16L‐dependent autophagy deficiency fostered the progression of EndMT and renal fibrosis after kidney transplantation.^12^ Furthermore, we discovered that everolimus was capable to restoring autophagic flux, thus diminishing renal allograft interstitial fibrosis via the inhibition of the mTOR pathway.^45^ In our study, we affirmed that BNIP3 knockdown negates the augmented mitophagy, and reverses the amelioration in ECM deposition and EndMT induced by *Rictor* ablation in endothelial cells after kidney transplantation. Furthermore, Rictor/mTORC2 signalling is regarded as a negative regulator of autophagy and mitophagy, exerting its inhibitory effects through the phosphorylation of Akt at Ser473,^27^ PKCα,^46^ or SGK1.^28^, ^47^ Our observations indicated that *Rictor* gene knockout resulted in diminished phosphorylation of downstream substrates, including Akt, PKCα, and SGK1. Consequently, we propose that these substrates of the Rictor/mTORC2 pathway might collectively influence the regulation of BNIP3‐mediated mitophagy.

In this investigation, we also predicted and subsequently verified the ubiquitination of BNIP3 at the K130 site. Furthermore, our observations revealed that Rictor/mTORC2 signalling orchestrated the proteasome‐dependent degradation of BNIP3 at K130 via K48‐linked ubiquitination. The posttranslational modifications of BNIP3 play a pivotal role in mitophagy regulation. Cao Yu et al.,^48^ for instance, elucidated that FBXL4 constitutes an SCF‐FBXL4 ubiquitin E3 ligase complex, facilitating the ubiquitination and subsequent degradation of BNIP3. Moreover, phosphorylation of BNIP3 is integral to mitophagy regulation. During hypoxic conditions, JNK1/2‐mediated phosphorylation at Ser 60/Thr 66 of BNIP3 amplified mitophagy, curtailing the proteasome‐dependent degradation of BNIP3.^49^ In contrast, BNIP3 dephosphorylation by PP1 and PP2A hampered mitophagy, accelerating BNIP3 degradation via the ubiquitin‐proteasome system.^49^ Although we identify MARCH5 as the specifical E3 ligase regulating BNIP3 degradation, it fails to pinpoint the specific modulation mechanism of MARCH5 by Rictor/mTORC2 signalling. Further studies are still required to elucidate that for a comprehensive understanding of Rictor‐mediated mitophagy.

To summarize, this study demonstrates the substantial role of Rictor/mTORC2 signalling in driving the progression of renal vascular EndMT and allograft interstitial fibrosis through the modulation of BNIP3‐mediated mitophagy. These results might unveil new perspectives and potential therapeutic interventions for preventing the progression of renal allograft interstitial fibrosis.

## AUTHOR CONTRIBUTIONS

Ruoyun Tan and Min Gu designed the study; Dengyuan Feng, Zeping Gui, Zhen Xu, Jianjian Zhang, and Bin Ni performed the experiments; Dengyuan Feng, Zeping Gui, Jiawen Liu, Shuang Fei, Hao Chen, Li Sun, and Ruoyun Tan acquired and analyzed the data; Dengyuan Feng and Ruoyun Tan wrote the paper; Zijie Wang and Min Gu revised the manuscript. All authors read and approved the final manuscript.

## CONFLICT OF INTEREST STATEMENT

The authors declare no conflict of interest.

## FUNDING INFORMATION

This project was supported by the National Natural Science Foundation of China (grant no.: 82070769, 82270790, 81900684, 81870512, 81770751), “333 High Level Talents Project” in Jiangsu Province (grant no.: BRA2017532, BRA2016514).

## DATA SHARING STATEMENT

All relevant data are available in the figures and supplementary materials. Any additional information required is available from the corresponding authors.

## ETHICAL APPROVAL

The studies involving human participants were approved by the Ethics Committee of the First Affiliated Hospital of Nanjing Medical University (approval number: 2023‐SRFA‐007). All participants signed informed consent. All animal experiments were conducted according to protocols approved by the Animal Research Ethics Committee of Nanjing Medical University (approval number: IACUC‐2109025).

## Supporting information

Supporting Information

Supporting Information
