# Supplementary material for: Rictor/mTORC2 signalling contributes to renal vascular endothelial‐to‐mesenchymal transition and renal allograft interstitial fibrosis by regulating BNIP3‐mediated mitophagy
Source: Clin Transl Med. 2024 May 20;14(5):e1686. doi: 10.1002/ctm2.1686 (PMC11106512; doi:10.1002/ctm2.1686)
Supplement: Supplementary file 1 — Supporting Information [file CTM2-14-e1686-s001.docx]

**Supplementary Table 1: The antibodies used in this study.**

| **Antibodies for western blotting** | **Company** | **Identifier** |
| --- | --- | --- |
| Rictor | Cell Signaling Technology | #2114 |
| p-Akt (Ser473) | Proteintech | 80455-1-RR |
| p-Akt (Thr308) | Affinity | AF3262 |
| p-PKCα | HUABIO | ET1702-17 |
| p-SGK1 | Affinity | AF3001 |
| CD31 | Cell Signaling Technology | #77699 |
| Fibronectin | Proteintech | 15613-1-AP |
| Collagen І | ABclonal | A1352 |
| αSMA | Cell Signaling Technology | #19245 |
| LC3 | Cell Signaling Technology | #3868 |
| TOMM20 | Proteintech | 66777-1-Ig |
| VDAC1 | Proteintech | 55259-1-AP |
| TIM23 | Proteintech | 11123-1-AP |
| PINK1 | Cell Signaling Technology | #6946 |
| NIX | Cell Signaling Technology | #12396 |
| FUNDC1 | ABclonal | A16318 |
| BNIP3 | Cell Signaling Technology | #44060 |
| BNIP3 | Proteintech | 68091-1-Ig |
| Ubiquitin | Santa Cruz | sc-8017 |
| Flag | Cell Signaling Technology | 14793 |
| HA | Proteintech | 51064-2-AP |
| Myc | Santa Cruz | sc-40 |
| FSP1 | Proteintech | 16105-1-AP |
| MARCH5 | Proteintech | 12213-1-AP |
| GAPDH | Proteintech | 60004-1-Ig |
| β-ACTIN | Proteintech | 66009-1-Ig |
| Goat Anti-Mouse IgG | Proteintech | SA00001-1 |
| Goat Anti-Rabbit IgG | Proteintech | SA00001-2 |

| **Antibodies for immunofluorescence** | **Company** | **Identifier** |
| --- | --- | --- |
| CD31 | R&D Systems | AF3628 |
| Rictor | Abcam | ab104838 |
| BNIP3 | Cell Signaling Technology | #44060 |
| LC3 | Cell Signaling Technology | #3868 |
| Cy™3 AffiniPure Goat Anti-Rabbit IgG | Jackson ImmunoResearch | 111-165-003 |
| Fluorescein (FITC) AffiniPure Goat Anti-Mouse IgG | Jackson ImmunoResearch | 115-095-003 |
| Cy3-labeled Donkey Anti-Goat IgG | Beyotime | A0502 |
| Fluorescein (FITC) AffiniPure Donkey Anti-Rabit IgG (H+L) | Jackson ImmunoResearch | 711-095-152 |
