# Supplementary material for: Rictor/mTORC2 signalling contributes to renal vascular endothelial‐to‐mesenchymal transition and renal allograft interstitial fibrosis by regulating BNIP3‐mediated mitophagy
Source: Clin Transl Med. 2024 May 20;14(5):e1686. doi: 10.1002/ctm2.1686 (PMC11106512; doi:10.1002/ctm2.1686)
Supplement: Supplementary file 2 — Supporting Information [file CTM2-14-e1686-s002.docx]

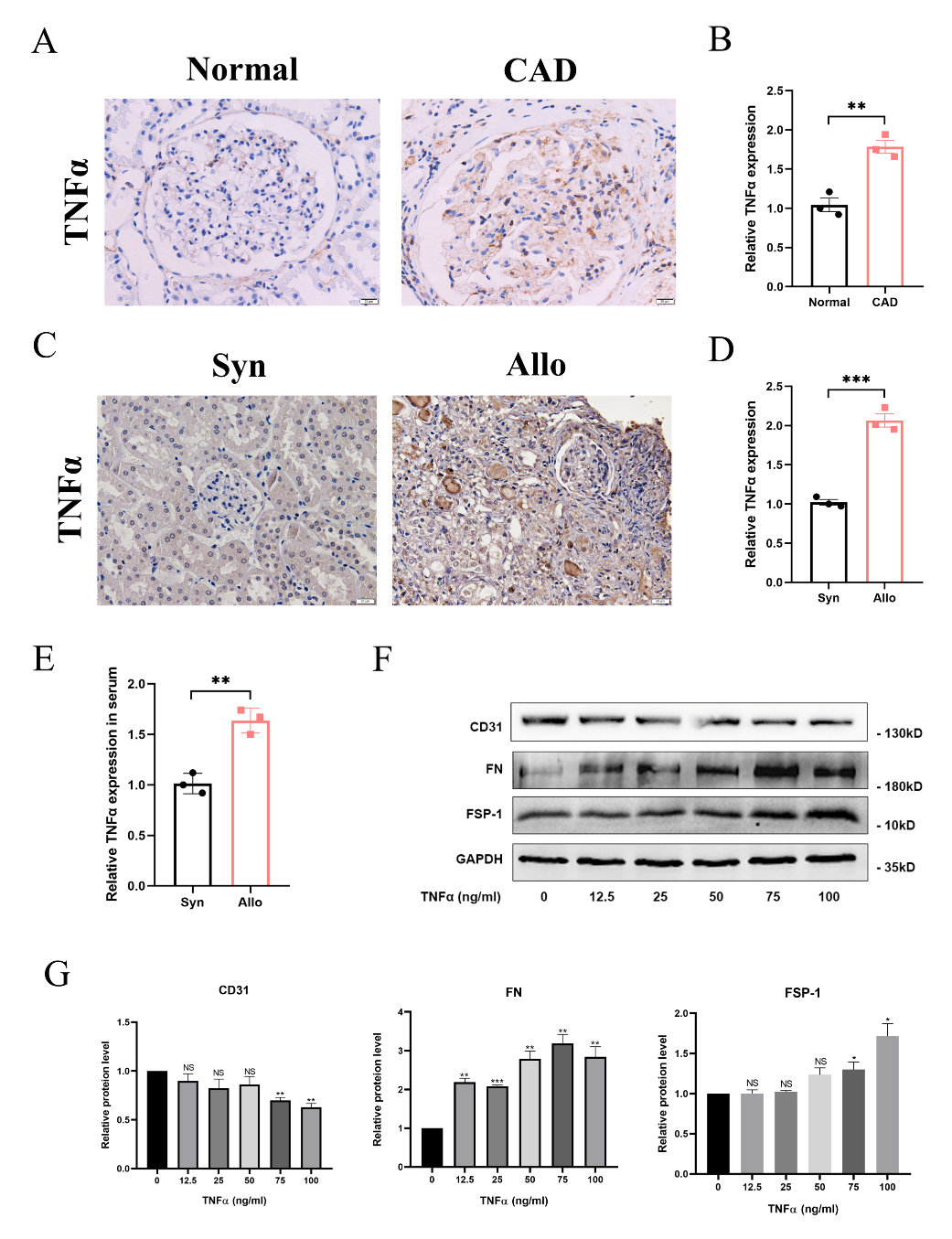


**Supplementary Figure 1: TNFα expression is increased in chronic renal allograft dysfunction (CAD) patients and renal allogeneic transplanted mice and TNFα contributes to the formation of EndMT.**

**A, B**. Representative IHC images (A) and statistical graph of semi-quantitative analysis (B) of TNFα in kidney tissues from the normal and CAD patients. (n = 3. Bar = 20μm). **C, D**. Representative IHC images (C) and statistical graph of semi-quantitative analysis(D) of TNFα in transplanted kidney tissues from the Syn and Allo mice. (n = 3. Bar = 20μm). **E**. The results of quantitative ELISA-based chemiluminescent assay of TNFα abundance in serum from the Syn and Allo mice. (n = 3). **F, G**: The results of western blot analyses (F) and quantitative analyses of the relative abundance (G) of FN, FSP-1, and GAPDH expression in HUVECs treated with different concentration of TNFα for 48 hours. **p* < 0.05, ***p* < 0.01, ****p* < 0.001.


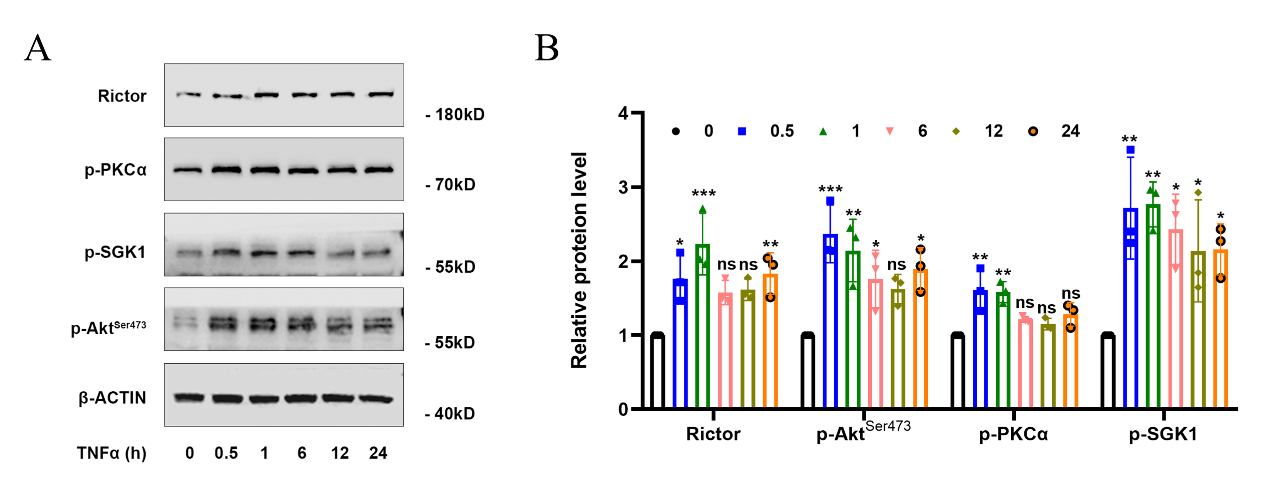


**Supplementary Figure 2: TNFα treatment induces the Rictor/mTORC2 signaling activation in HUVECs**

**A, B**: The results of western blot analyses (A) and quantitative analyses(B) of the relative abundance of Rictor, p-PKCα, p-SGK1, and p-Akt^Ser473^ in HUVECs treated with 100ng/ml TNFα for different peroid. **p* < 0.05, ***p* < 0.01, ****p* < 0.001.


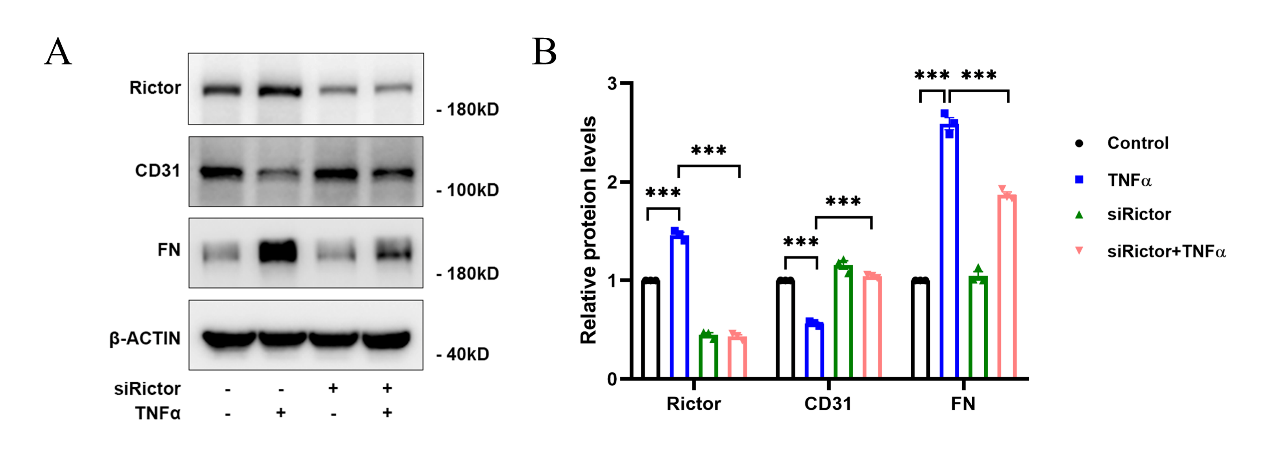


**Supplementary Figure 3: Rictor knockdown alleviated TNFα-induced EndMT in the human renal glomerular endothelial cells.**

**A, B**: The results of western blot analyses (A) and quantitative analyses of the relative abundance (B) of Rictor, CD31, and FN expression in the human renal glomerular endothelial cells transfected with siNC or siRictor. Data were presented as mean ± SEM. ***p < 0.001.


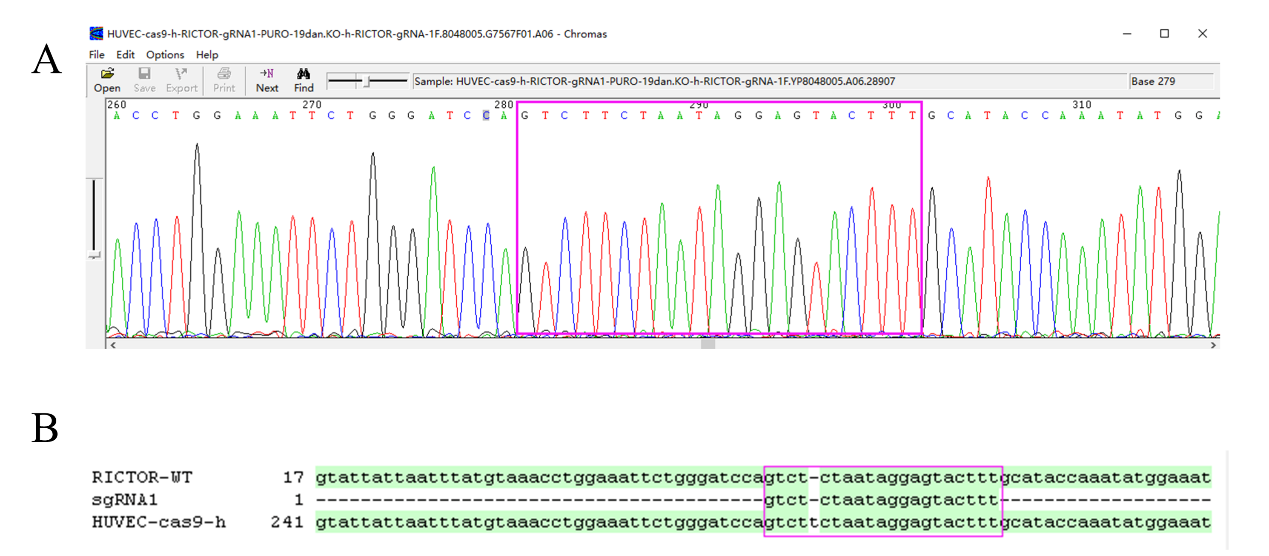


**Supplementary Figure 4: *Rictor* knockout (KO) HUVEC line is generated using CRISPR/Cas9-mediated gene editing.**

**A**: Monoclonal peak diagram of Rictor Cas9-gRNA: The Rictor mutation region is selected with a pink box. **B**: The monoclonal sequencing is compared with the wild-type sequence, as shown in the figure below: the pink box selects the mutation site, and the double underline is the sgRNA1 target sequence.
